# Deleting Titin’s C-Terminal PEVK Exons Increases Passive Stiffness, Alters Splicing, and Induces Cross-Sectional and Longitudinal Hypertrophy in Skeletal Muscle

**DOI:** 10.3389/fphys.2020.00494

**Published:** 2020-05-29

**Authors:** Robbert J. van der Pijl, Brian Hudson, Tomotaroh Granzier-Nakajima, Frank Li, Anne M. Knottnerus, John Smith, Charles S. Chung, Michael Gotthardt, Henk L. Granzier, Coen A. C. Ottenheijm

**Affiliations:** ^1^Cellular and Molecular Medicine, University of Arizona, Tucson, AZ, United States; ^2^Department of Physiology, Amsterdam UMC, Amsterdam, Netherlands; ^3^Department of Physiology, Wayne State University, Detroit, MI, United States; ^4^Max-Delbruck-Center for Molecular Medicine, Berlin, Germany; ^5^Cardiology, Virchow Klinikum, Charité University Medicine, Berlin, Germany

**Keywords:** passive tension, titin, PEVK region, hypertrophy, RNA splicing

## Abstract

The Proline, Glutamate, Valine and Lysine-rich (PEVK) region of titin constitutes an entropic spring that provides passive tension to striated muscle. To study the functional and structural repercussions of a small reduction in the size of the PEVK region, we investigated skeletal muscles of a mouse with the constitutively expressed C-terminal PEVK exons 219–225 deleted, the Ttn^Δ219–225^ model (MGI: Ttn^TM 2.1*Mgot*^). Based on this deletion, passive tension in skeletal muscle was predicted to be increased by ∼17% (sarcomere length 3.0 μm). In contrast, measured passive tension (sarcomere length 3.0 μm) in both soleus and EDL muscles was increased 53 ± 11% and 62 ± 4%, respectively. This unexpected increase was due to changes in titin, not to alterations in the extracellular matrix, and is likely caused by co-expression of two titin isoforms in Ttn^Δ219–225^ muscles: a larger isoform that represents the Ttn^Δ219–225^ N2A titin and a smaller isoform, referred to as N2A2. N2A2 represents a splicing adaption with reduced expression of spring element exons, as determined by titin exon microarray analysis. Maximal tetanic tension was increased in Ttn^Δ219–225^ soleus muscle (WT 240 ± 9; Ttn^Δ219–225^ 276 ± 17 mN/mm^2^), but was reduced in EDL muscle (WT 315 ± 9; Ttn^Δ219–225^ 280 ± 14 mN/mm^2^). The changes in active tension coincided with a switch toward slow fiber types and, unexpectedly, faster kinetics of tension generation and relaxation. Functional overload (FO; ablation) and hindlimb suspension (HS; unloading) experiments were also conducted. Ttn^Δ219–225^ mice showed increases in both longitudinal hypertrophy (increased number of sarcomeres in series) and cross-sectional hypertrophy (increased number of sarcomeres in parallel) in response to FO and attenuated cross-sectional atrophy in response to HS. In summary, slow- and fast-twitch muscles in a mouse model devoid of titin’s PEVK exons 219–225 have high passive tension, due in part to alterations elsewhere in splicing of titin’s spring region, increased kinetics of tension generation and relaxation, and altered trophic responses to both functional overload and unloading. This implicates titin’s C-terminal PEVK region in regulating passive and active muscle mechanics and muscle plasticity.

## Introduction

Titin is a giant protein located inside the striated muscle sarcomere where it anchors inside the Z-disk and M-band and spans the half-sarcomere. This layout in the sarcomere uniquely positions titin to act as a molecular spring (Labeit and Kolmerer, 1995). The spring function of titin is primarily regulated through elastic domains in the I-band region of the sarcomere. In skeletal muscle, these domains consist of serially linked immunoglobulin(Ig)-like repeats and a spring element consisting of the PEVK (Proline, Glutamate, Valine, Lysine-rich) region (Watanabe et al., 2002; Nagy et al., 2005). Titin’s spring-like behavior, combined with its contiguous arrangement, provides passive tension to skeletal muscle. Indeed, complete removal of titin results in loss of sarcomere structure and passive and active tension (Horowits et al., 1986; Radke et al., 2019), supporting titin’s vital role in muscle mechanics and sarcomerogenesis.

The role of titin in passive tension has been extensively studied in the heart [reviewed in Granzier and Irving (1995); Linke and Fernandez (2002), and LeWinter et al. (2007)], with several studies targeting titin-stiffness for therapeutic benefit in cardiac dysfunction (Hinze et al., 2016; Methawasin et al., 2016). Deletion models such as the Ttn^Δ30–38^ [proximal tandem Ig segment; Mouse genome informatics entry (MGI): Ttn^TM 1.1*Hgra*^ (Chung et al., 2013)], Ttn^Δ49^ [N2B; MGI: Ttn^TM 1*Mgot*^ (Radke et al., 2007)] and Ttn^Δ251–269^ [I/A-junction; MGI: Ttn^TM 2.1*Hgra*^ (Granzier et al., 2014)] models show increased passive tension in the heart, whereas the deletion of Rbm20^Δ*RRM*^ [Rbm20 RNA recognition motif deletion; MGI: Rbm20^TM 1*Hgra*^ (Guo et al., 2012; Methawasin et al., 2014)], involved in titin RNA-splicing, results in more compliant titin isoforms and reduced passive tension. Trophic responses to these deletions are varied, with Ttn^Δ251–269^ and Ttn^Δ219–225^ hearts developing mild hypertrophy, and Ttn^Δ30–38^ and Ttn^Δ49^ hearts showing atrophy. Thus, the varied responses to altering titin stiffness in the heart exemplify the complexity of the mechanisms underlying passive tension and muscle trophicity.

In skeletal muscles, the effect of modifying titin stiffness is even more complex. Skeletal muscles possess highly variably spliced tandem Ig segments and PEVK regions (Li et al., 2012; Savarese et al., 2018), with fetal muscle possessing the highest inclusion of exons (Lahmers et al., 2004). This variable titin splicing in skeletal muscles makes studying deletions in titin more challenging, as the deletions are likely to have variable effects on muscle structure and function; effects that are likely specific to muscle types. The splicing of titin is controlled by RNA-binding protein 20 (Rbm20). Both rat and mouse models of complete or partial ablation of the Rbm20 protein result in titin isoforms of 3.8–3.9 mDa in skeletal muscles, with reduced passive tension (Mateja et al., 2013; van der Pijl et al., 2018). Whereas Rbm20 animal models indirectly affect the PEVK region of titin, there are two mouse models that specifically target this region: the Ttn^Δ112–158^ [MGI; Ttn^TM 4.1*Hgra*^ (Brynnel et al., 2018)] and Ttn^Δ219–225^ [N2B PEVK KO; MGI: Ttn^TM 2.1*Mgot*^ (Granzier et al., 2009)] deletion models. The Ttn^Δ112–158^ model targets the N-terminal portion of the PEVK region, deleting up to 47 of 100 PEVK repeats found in the mouse gene (Granzier et al., 2007). Ttn^Δ112–158^ mice showed marked increases in passive stiffness of skeletal muscle. In response, muscles developed longitudinal hypertrophy, i.e. serial growth of the number of sarcomeres, to reduce the sarcomere length working range and the apparent passive muscle stiffness. The Ttn^Δ219–225^ model targets only a small portion of the PEVK region, the constitutively expressed seven C-terminal PEVK exons. This model was designed to target the only PEVK repeats expressed in the N2B cardiac titin isoform. In the heart, this mouse displays increased passive tension and hypertrophy. The structure and function of skeletal muscles in this model has not been investigated. Considering the relatively small size of the deletion of the Ttn^Δ219–225^ model, we hypothesized that the deletion would minimally affect passive tension in skeletal muscle, allowing us to study titin stiffness in relation to muscle function in the absence of trophic remodeling.

Our findings revealed that the Ttn^Δ219–225^ soleus and extensor digitorum longus (EDL) muscles have increased passive tension, partly due to the appearance of a smaller secondary titin isoform. Contrary to our hypothesis, the increased passive tension induced both cross-sectional and longitudinal hypertrophy. Active tensions were higher in soleus muscle, but not in the EDL, while the kinetics of force generation and relaxation were faster. Finally, Ttn^Δ219–225^ muscles displayed a “sensitization” to hypertrophic stimuli. These findings indicate that a small truncation in titin’s PEVK region can have substantial repercussions for the structure and function of skeletal muscle.

## Materials and Methods

### Animals and Tissue Collection

Ttn^Δ219–225^ mouse model generation was previously described by Granzier et al. (Granzier et al., 2009) and the Rbm20^Δ*RRM*^ mouse model has previously been described by Methawasin et al. (2014). Mice were maintained on a mixed C57BL/6 × 129S6 background, on a heterozygous to heterozygous breeding scheme. Age-matched homozygous Ttn^Δ219–225^ (Δ) and wildtype littermates were used for experiments. Mouse soleus (SOL), extensor digitorum longus (EDL), tibialis cranialis (TC), and diaphragm (DIA) were collected from full grown 3.5-month-old male mice and to show progressive changes in muscle mass, 6-month old male mice were also used. A subset of the experiments was also performed on female mice of both genotypes, to study whether the results obtained were gender-dependent. Prior to starting experiments, mice were anesthetized with isoflurane and sacrificed by cervical dislocation. Tissues were rapidly excised and the muscles dissected in oxygenated HEPES solution (NaCl 133.5 mM; KCl 5 mM; NaH_2_PO_4_ 1.2 mM; MgSO_4_ 1.2 mM; HEPES 10 mM). Dissected muscles were either: (1) snap frozen in liquid nitrogen, (2) used directly for intact muscle contractility studies, or (3) chemically skinned in relaxing solution (BES 40 mM, EGTA 10 mM, MgCl_2_ 6.56 mM, ATP 5.88 mM, DTT 1 mM, K-propionate 46.35 mM, creatine phosphate 15 mM, pH 7.0) with 1% Triton-X-100, overnight at 4°C, washed thoroughly with relaxing solution, and stored up to 1 month at −20° in relaxing solution containing 50% (v/v) glycerol. To prevent protein degradation, all solutions contained protease inhibitors (phenylmethylsulfonyl fluoride (PMSF) 0.5 mM; Leupeptin 0.04 mM; E64 0.01 mM). All animal experiments were approved by the University of Arizona Institutional Animal Care and Use Committee and followed the U.S. National Institutes of Health “Using Animals in Intramural Research” guidelines for animal use.

### Functional Overload Study

Male 3.5-month-old mice were sedated with 1–3% isoflurane and underwent bilateral ablation of the gastrocnemius and plantaris muscles to induce functional overload (FO) hypertrophy in soleus muscle. Briefly, hair was removed from the lower leg, with depilatory cream and cleaned with alternating scrubs of 70% ethanol and betadine. A longitudinal incision was made to reveal the gastrocnemius-complex and the Achilles tendon. The soleus tendon was carefully isolated and the remaining portion of the Achilles tendon was cut. The gastrocnemius and planteris were gently blunt-dissected away from the soleus muscle, until approximately 75% was free and the muscles were then removed and the skin sutured together. Mice were allowed to recover and then freely move in their cage for 10 days after which they were sacrificed for subsequent studies.

### Hindlimb Suspension Study

Male 3.5-month-old mice were suspended by the tail to induce atrophy of the hindlimb muscles, as previously described (Labeit et al., 2010). Briefly, the tail of each mouse was placed in a harness, which is used to elevate the pelvis so that the mice are angled ∼45°, with the feet of the hindlimbs unable to contact the cage floor. The suspended mice were housed in custom-designed cages where they could freely move around on their front limbs, with free access to water and food. Mice were suspended for 10 days before being sacrificed for subsequent studies.

### Titin Microarray Studies

The microarray experiments were performed as described previously (Buck et al., 2010). Briefly, freshly dissected soleus and EDL were stored in RNA*later* (Ambion) and RNA was subsequently isolated using the RNeasy Fibrous Tissue Mini Kit (Qiagen). RNA was amplified using the SenseAmp kit (Genisphere) and Superscript III reverse transcriptase enzyme (Invitrogen). Reverse transcription and dye coupling was done using superscript plus indirect cDNA labeling module (Invitrogen). WT and Ttn^Δ219–225^ samples were reciprocally labeled with Alexa Fluor 555 or Alexa Fluor 647, and samples of both genotypes were cohybridized on a custom microarray (Lahmers et al., 2004). For each sample 750 ng of cDNA (Nanodrop, Thermo Fisher Scientific) was hybridized with Slide-Hyb buffer #1 (Ambion) for 16 h at 42°C and continuously washed using a GeneTac Hybridization Station (Genomic Solutions). Microarrays were scanned at 595 and 685 nm with an Array WoRx scanner, spot-finding with SoftWoRx Tracker, and analysis using the R module, CARMA (Greer et al., 2006). The analysis detects relative changes in the fluorescence of a probe, represented as a fold-difference between WT and Ttn^Δ219–225^ exon expression. A positive fold change indicates an increase in expression and a negative fold-change indicates a decrease in expression compared to WT samples. Eight samples of each muscle and genotype, consisting of four males and four females each, were run to determine the average fold-change in the Ttn^Δ219–225^ mice.

### Gel Electrophoresis

SDS-agarose gel electrophoresis (SDS-AGE) was performed as previously described (Warren et al., 2003). Briefly, muscle samples were solubilized in a urea and glycerol buffer (with protease inhibitors Leupeptin 0.04 mM; E64 0.16 mM; and PMSF 0.2 mM). The gels were run at 15 mA per gel for 3 h and 20 min in a SE600X vertical gel system (Hoefer Inc., United States), stained with Neuhoff’s coomassie, and subsequently scanned and analyzed using One-D scan EX (Scanalytics Inc., United States) software. The integrated optical density of titin and myosin heavy chain (MyHC) was determined as a function of the slope of the linear range between integrated optical density and loaded volume. Note that N2A and N2A2 Gaussians fits do not fully separate, leading to a slight overestimation of titin levels in the Ttn^Δ219–225^ muscles. SDS-PAGE gels for MyHC separation were performed as previously described (Agbulut et al., 1996). MyHC IIa overlaps with IIx, therefore we refer to this band as IIa/x.

### Simulated Force

To estimate the effect of the exon 219–225 deletion on titin-based passive force we determined the force-sarcomere length relation of a single titin molecule in the sarcomere with and without exons 219–225. These calculation take into account the extensibility of the various titin segments, in the absence of post-translational modifications. The spring region of titin was simulated as two WLCs in series: (1) the tandem Ig segment (combined proximal and distal segments), and (2) the PEVK segment. For a WLC, the external force is given by equation 1.

(1) $$F=\frac{k_{B}\,T}{L_{p}}\,\left(\frac{1}{4\,{\left(1-\frac{z}{L_{c}}\right)}^{2}}-\frac{1}{4}+\frac{z}{L_{c}}\right)$$

F is force (pN), Lp is the persistence length (nm), z is end-to-end extension (nm), Lc is the contour length (nm), k_*B*_ is Boltzmann’s constant, and T is absolute temperature. Based on previous work the PEVK segment was assumed to contain 1613 residues in WT mouse soleus muscle (Brynnel et al., 2018) and 190 residues less in Ttn^Δ219–225^ mouse soleus (Granzier et al., 2009). Assuming a maximal residue spacing of 0.38 nm per residue (Kellermayer et al., 1997; Anderson and Granzier, 2012), resulting in a PEVK Lc of 613 nm in WT and 541 nm in Ttn^Δ219–225^ mice. Tandem Ig segments were assumed to contain 90 Ig domains in both WT and Ttn^Δ219–225^ mice (Brynnel et al., 2018) with an average maximal spacing of folded Ig domains of 5 nm (Trombitas et al., 1998), resulting in a Lc of 450 nm. The persistence length (Lp) of the PEVK and tandem Ig segments were (based on previous work) assumed to be 1.0 nm and 10 nm, respectively, (Watanabe et al., 2002; Anderson and Granzier, 2012). Because spring elements are in series, they bear equivalent forces, and the fractional extension (z/L) at each force can therefore be calculated. Using the fractional extensions and the Lc values (above) the corresponding SLs were determined assuming that the inextensible Z-disk and A-band segments of titin are 700 nm long (per half sarcomere) (Trombitas et al., 1998).

### Measurement of Passive Tension

Small muscle strips (100–200 μm in diameter, ∼2 mm in length) were dissected from chemically skinned skeletal muscle preparations that had been stored in 50% glycerol/relaxing solution. Passive force was measured with a strain gauge force transducer and fiber length was controlled by a high-speed motor. Muscle strips were attached to the motor arm and the force transducer via aluminum T-clips and then lowered into a chamber containing relaxing solution (room temperature). The width and depth of the bundles were measured and the cross-sectional area (CSA) was calculated assuming an elliptical shape. The measured forces were then converted to tension (force/CSA). Sarcomere length (SL) was measured on-line by laser diffraction. At the beginning of each experiment, muscles were activated at SL 2.4 μm (pCa 4.0) to measure active tension. Typically, maximal activate tension was at least 100 mN/mm^2^, indicating normal myofilament function.

Fibers bathed in relaxing solution were stretched (10%/sec) from their slack length (2.0 μm) up to a SL of 3.0 μm, in 0.1 μm increments, followed by a 90 s hold. At the end of the hold phase the fiber was released back to slack length and allowed to rest 12 min before the next stretch. To determine titin and collagen contribution to passive force, thick and thin filaments were extracted with KCl/KI (KCl 0.6 mM and KI 1 mM, respectively), from the sarcomere, removing titin’s anchors. The remaining force, assumed to be collagen-based, was subtracted from the pre-extraction forces to determine titin-specific forces (Brynnel et al., 2018).

### Intact Muscle Contractility Experiments

For intact muscle experiments, fresh soleus and EDL muscles were used for their distinct tendons that make mechanical experiments possible and well known attributes as slow-twitch and fast-twitch muscles, respectively. Muscles were quickly dissected and, using silk suture, mounted vertically in a tissue bath between a dual-mode lever arm and a fixed hook (1200A Intact Muscle Test System, Aurora Scientific Inc., Canada). The muscle was bathed in continuously oxygenated (95% O_2_–5% CO_2_) mammalian Ringer solution (pH 7.4) and kept at a constant temperature (30°C) for the duration of the experiment. The muscle was stimulated directly with platinum plate electrodes placed in close apposition to the muscle. Muscle preload force was adjusted until optimal fiber length (L_0_) for maximal twitch force was achieved (pulse width of 200 ms).

#### Force-Frequency Protocol

Five minutes after completion of the passive tension protocol, the muscle was stimulated at various incremental frequencies (1, 5, 10, 20, 40, 60, 80, 100, 150, and 200 Hz). Stimuli were applied with a train duration of 400 ms and a 90 s interval. After completion of the contractility measurements, the weight of the muscle was determined. Cross-sectional area (in mm^2^) of the EDL and Soleus muscles was calculated using: CSA [cm^2^] = (muscle mass [g] × cos [θ])/(ρ [g × cm^–3^] × fiber length [cm]) (θ is the pennation angle and ρ is the physiological density of muscle) (Lieber and Ward, 2011).

A subset of these muscles was fixed at L_0_ by overnight immersion in 4% formaldehyde/1% glutaraldehyde. Intrinsic shrinkage was minimized by pinning the tissue during fixation. Thin full-length (tendon-tendon) fiber bundles were dissected from the fixed muscles and sarcomere length was measured by laser diffraction. The number of serial sarcomeres was calculated by dividing the length of the fiber bundles by the measured sarcomere length.

### Western Blots

Samples were run on 0.8% SDS-AGE gels for titin or 12% SDS-PAGE for signaling proteins, and transferred to PVDF membrane using a semi dry transfer unit (Bio-Rad; United States). The blots were stained with Ponceau S to visualize total transferred protein. The blots were then probed with CSRP3/MLP, FHL1, MARP1, MARP2, Ttn C-term, Ttn N-term, Erk, Thr202/Tyr204 p-Erk, mTOR, S2481 p-mTOR, and GAPDH (see antibody table for details). Secondary antibodies conjugated with fluorescent dyes with near-infrared excitation spectra were used for detection. Two-color IR western blots were scanned (Odyssey Infrared Imaging System, Li-Cor Biosciences, United States) and the images analyzed with Image studio light (Li-Cor Biosciences). All proteins were normalized against GAPDH, and, subsequently, (for FO and HS samples) normalized to control samples (EDL or soleus) to determine relative changes in protein quantity.

### Statistics

Data are presented as mean ± SEM. Significance was defined *p* < 0.05 as indicated in the figures. Statistical testing was performed using student *t*-test, one or two-way ANOVA as applicable. A linear model that accounts for experimental variability was used to analyze the microarray data. Curves were fitted for non-linear regression to determine the difference in fit between equations, an extra sum of squares F-test determined whether the data sets differed from each other.

## Results

### Titin-Based Passive Tension of Ttn^Δ219–225^ Is Disproportionately Increased

**FIGURE 1:**
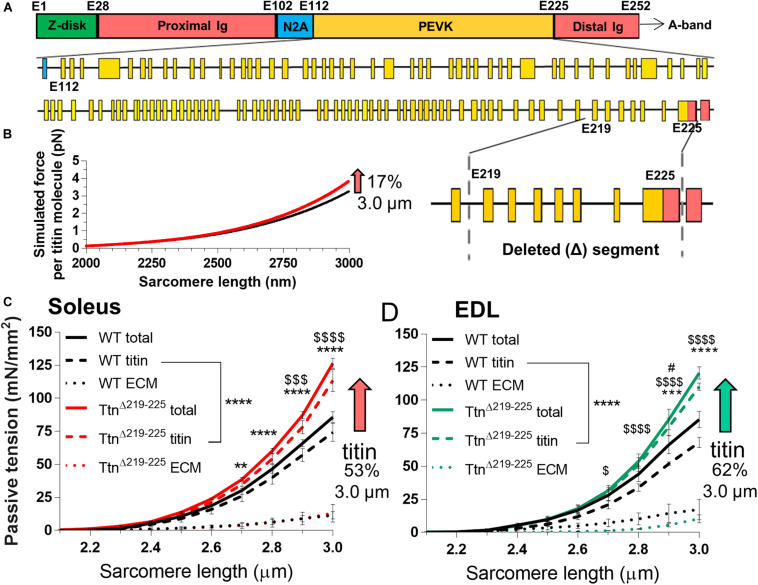
Passive tension in Ttn^Δ219–225^ mice. **(A)** Schematic of titin’s I-band region, with exon composition of PEVK segment (below) and detailed facet of deleted segment (bottom-right), (Yellow: PEVK exons; red: Ig domains, blue: unique sequence). **(B)** Simulated titin tension for wildtype (WT; black line) and Ttn^Δ219–225^ (red line), showing a minimal increase in passive tension (17% at SL 3.0 μm). The increase in passive tension was substantially higher in skinned soleus **(C**; 53%) and EDL **(D**; 62% at SL 3.0 μm). The increase in tension is due to changes in titin-based passive tension (curve fit *p* < 0.0001) and minor changes in extracellular matrix (ECM; *n* = 4 mice; *t*-test *p*-value: *total passive tension, $titin-based passive tension, ^#^ECM-based tension; *p < 0.05, **p < 0.01, ***p < 0.001, ****p < 0.0001).

Considering that the deleted exons 219–225 comprise a small fraction of the PEVK region of the titin gene (Figure 1A), the expected level of passive tension increase in Ttn^Δ219–225^ skeletal muscle was assessed first. Calculations of the force per titin molecule using a worm-line chain model (see section “Materials and Methods”), showed a small predicted passive tension increase (Figure 1B) that within the 2.3-3.0 μm SL range [selected because it includes the physiological SL range (Burkholder and Lieber, 2001)] was on average 9.7% (17% at SL: 3.0 μm). Passive tension was measured next, using demembranated fiber bundles of soleus and extensor digitorum longus (EDL) muscles (Figures 1C,D). Soleus muscle displayed an increase in passive tension of 36 ± 2% and EDL 27 ± 10% (SL range 2.3–3.0 μm) and a level of 48 ± 5% and 41 ± 5% at SL 3.0 μm, i.e. ∼2-fold higher than titin-based passive tension predicted. Because changes in the extracellular matrix (ECM) also contribute to passive tension (Trombitas et al., 2003; Chung et al., 2011) this larger than expected measured passive tension could be due to an adaption in the ECM. To test this, passive tension was measured both before and after extraction of the thin and thick filaments (see section “Materials and Methods”), thereby removing titin’s anchors in the sarcomere and abolishing titin-based passive tension (Wang et al., 1993; Wu et al., 2000). These experiments provided both the total tension (titin and ECM) and the ECM-based passive tension (extraction insensitive tension) and from this the titin-based fraction was calculated (total passive tension minus ECM-based passive stiffness). This showed that the ECM-based passive tension in WT and Ttn^Δ219–225^ muscles did not differ significantly but that titin-based tension solely accounted for the higher passive tension in Ttn^Δ219–225^ muscles: 52 ± 11% and 62 ± 4% for soleus and EDL muscle, respectively, (at SL 3.0 μm, Figures 1C,D, striped lines; titin-based passive tension, dotted lines; ECM-based passive tension). Thus, the large increase in passive tension in Ttn^Δ219–225^ muscles is likely titin-based and the increase is much higher than predicted based on the deletion of PEVK exons 219–225.

### Aberrant Titin Splicing in Skeletal Muscle of Ttn^Δ219–225^ Mice

**FIGURE 2:**
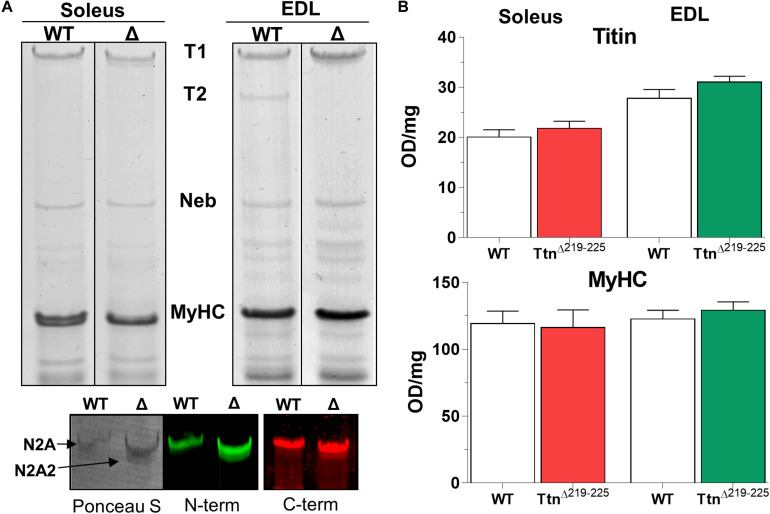
Titin in skeletal muscles of Ttn^Δ219–225^ mice. **(A)** Representative gel images of soleus (left) and EDL (right) Ttn^Δ219–225^ mice show a second band below the mutant N2A isoform, that is detected by both N- and C-terminal titin antibodies on Western blot, making it a full length titin isoform: N2A2 (left image: Ponceau S; middle: Z1–Z3 antibody, N-term titin. Right: M8M9 antibody, C-term titin). **(B)** Quantification of total titin (top graph) and myosin heavy chain (below), showing no changes in content between wildtype (WT) and Ttn^Δ219–225^ content. OD: optical density.

**FIGURE 3:**
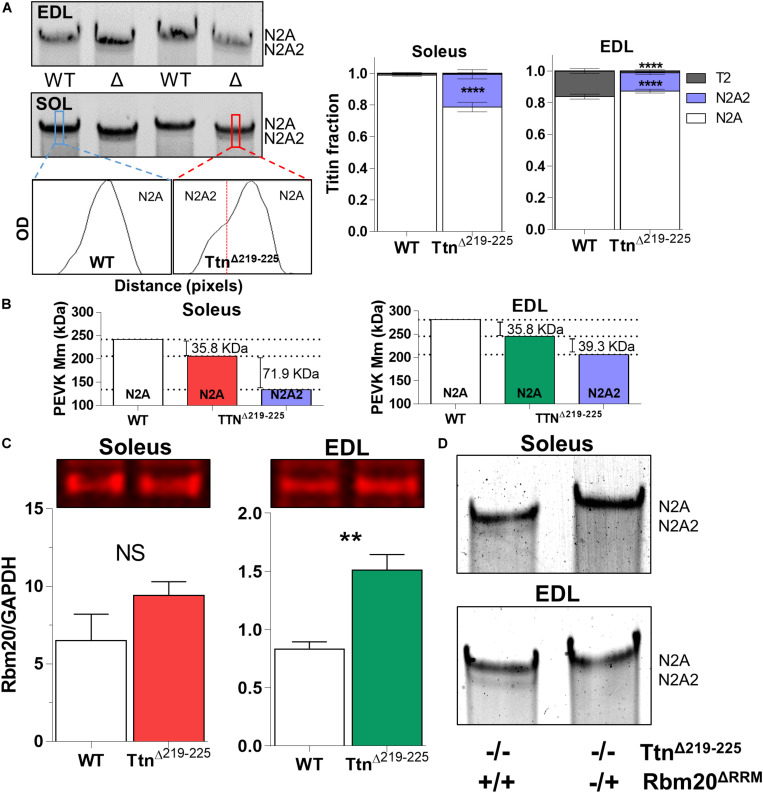
N2A2 titin is an alternative splice isoform, under control of Rbm20. **(A)** Representative detail gel images of EDL (top) and Soleus (middle), and densitometry profiles (below) of wildtype and Ttn^Δ219–225^ soleus muscle, showing the N2A2 isoform. Quantification of the N2A2 isoform as a ratio over total titin [TT; N2A, N2A2 and titin degradation products (T2)], showing 15-20% of total titin consists of N2A2 (****p < 0.0001 *t*-test). Soleus muscle also has a higher quantity (*p < 0.05, *t*-test) of N2A2 titin compared to EDL muscle. **(B)** Calculated effect of titin splicing on molecular mass of the PEVK-region of soleus (left) and EDL (right) muscle, as determined by custom titin gene array (*N* = 7–8 for each muscle; both female and male mice). Splicing in the I-band, showing additional splicing in primarily the PEVK region, A-band segment was unaffected (see Supplementary Table 1). **(C)** Western blots show an increase in Rbm20 levels in EDL (**p < 0.01, *t*-test) and a trending increase in soleus (*N* = 4–5 for each muscle). **(D)** Soleus and EDL muscles (*N* = 3) from homozygous (−/−) Ttn^Δ219–225^ mice crossed with heterozygous (±) Rbm20^Δ*RRM*^ (inactivated splice activity) mice, show that Rbm20 controls N2A2 expression.

To explain the higher than expected titin-based passive tension in the Ttn^Δ219–225^ muscles, we first performed SDS-AGE to assess titin at the protein level. Expression levels of titin and myosin heavy chain (MyHC) in Ttn^Δ219–225^ mice were not different in either soleus or EDL muscle (Figure 2). EDL muscle appears to contain more titin, compared to soleus muscle. As the current sarcomere models predict a defined number (6) of titin molecules per sarcomere (Granzier and Irving, 1995; Liversage et al., 2001), it is possible that EDL has a larger pool of unincorporated titin molecules. In Ttn^Δ219–225^ soleus and EDL muscles the presence of a second titin band was detected just below the N2A titin band, referred to as N2A2 (Figure 3A). We performed western blots to study whether this N2A2 band was either a truncation or a smaller full-length titin isoform. Both the titin N-term and the C-term antibodies stained this lower band (Figure 2A, Bottom panels), indicating that it represents a full-length titin isoform. Quantitative analysis showed that N2A2 is ∼20% of total titin in soleus muscle and ∼10% in EDL muscle (Figure 3A, right panels). To study N2A2 titin at the transcript level, custom titin exon microarrays were used to determine changes in exon inclusion in Ttn^Δ219–225^ muscles compared to wildtype (WT) mice. Results for both the soleus and EDL muscles confirmed the expected deletion of exons 219–225 and revealed additional splicing in primarily the PEVK region of titin (Supplementary Table 1). Changes in titin Z-disc splicing were also detected (consistent with fiber type switching, see below), as well as minor changes in the proximal Ig-segment, but exon expression was unaltered in the A-band segment of titin (Supplementary Table 1). The additional splicing in the PEVK segment is calculated to give a size shift of 71.5 kDa in soleus (Figure 3B, left) and 39.3 kDa in EDL titin (Figure 3B, right). SDS-AGE gels revealed comparable titin migration patterns between soleus and EDL muscles (Figure 3A), and showed that Ttn^Δ219–225^ soleus has more N2A2 (20.6 ± 2.9%; Figure 3A, right panels) compared to EDL (11.5 ± 1.0%). The low N2A2 transcript levels in EDL limited the detection capacity by the titin microarray, suggesting that the N2A2 isoform is likely similar between soleus and EDL. To illustrate the potential effect of the N2A2 isoform on passive tension, we repeated the WLC for N2A2 (Supplementary Figure 1). At 100% N2A2 expression levels the passive tension is substantially increased, whereas at 20% N2A2 expression levels (similar to soleus levels) passive tension is moderately increased. This supports the notion that N2A2 contributes to the higher than expected passive tension observed in Figures 1C,D. Subsequently, we explored whether Rbm20, a titin splice factor (Guo et al., 2012; Li et al., 2013; Buck et al., 2014), regulates the splicing of N2A2 titin. Western blot analyses revealed a trending increase in Rbm20 levels in Ttn^Δ219–225^ soleus (*P* = 0.11; Figure 3C; left panel) and significantly increased Rbm20 levels in EDL (*P* > 0.01; Figure 3C; right panel). To further establish that Rbm20 controls N2A2 splicing *in vivo*, we crossed the Ttn^Δ219–225^ (homozygous) mouse with the Rbm20^Δ*RRM*^ (heterozygous) mouse model and studied titin isoform expression. The data reveal that reducing Rbm20 levels results in soleus and EDL muscles with single mutant N2A isoforms (Figure 3D). This confirms that the aberrant splicing of titin in the Ttn^Δ219–225^ mouse model is due to altered regulation of Rbm20.

### Ttn^Δ219–225^ Muscles Display Changes in Active Tension, With Faster Tension Kinetics

**FIGURE 4:**
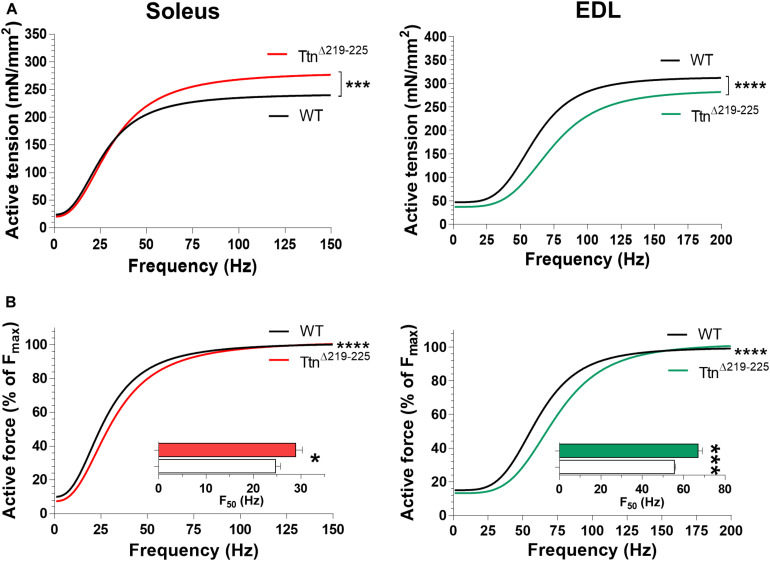
Muscle contractility of Ttn^Δ219–225^ mice. **(A)** curve fit of the active tension-stimulation frequency relation for soleus (left; WT, black line; Ttn^Δ219–225^ red line) and EDL (right; WT, black line; Ttn^Δ219–225^ green line), respectively, (*N* = 6–7, males; soleus maximal stimulation frequency: 150 Hz, and EDL: 200 Hz). **(B)** Curve fit in which active force is expressed as a % of maximum force for soleus (left) and EDL (right); insert depicts the frequency that produces 50% of the maximum active tension (F_50_). Curve fit; ***p < 0.001, ****p < 0.0001 and inserts *t*-test; *p < 0.05, ***p < 0.001.

To determine if the increased passive tension of Ttn^Δ219–225^ muscles affected active tension (Horowits and Podolsky, 1987), intact soleus and EDL muscles were electrically stimulated at L_0_, defined as the length of the muscle at which they produce the highest maximum tetanic force. Soleus muscle (Figure 4A, left) showed a pronounced increase in tetanic tension (240 ± 9 mN/mm^2^ in WT to 276 ± 17 mN/mm^2^; stimulation frequency 150 Hz), caused by hypertrophy (discussed below). In contrast, the EDL muscle (Figure 4A, right) showed a decrease in tetanic tension (315 ± 9 in WT to 280 ± 14 in Ttn^Δ219–225^; stimulation frequency 200 Hz). Thus, in the Ttn^Δ219–225^ mice, the maximal force generating capacity is differently affected in soleus than in EDL muscle.

The tension-frequency relations, when normalized to maximum force, of both soleus and EDL muscles from Ttn^Δ219–225^ mice were shifted rightward (Figure 4B). Consequently, the F_50_, the frequency at which the force is at 50% of maximum, was increased in both soleus (Figure 4B insert; 24.7 ± 1.0 Hz in WT to 28.9 ± 1.4 Hz in Ttn^Δ219–225^) and EDL (Figure 4B insert; 55.5 ± 0.6 Hz WT to 67.0 ± 2.0 Hz in Ttn^Δ219–225^). Tension-frequency relations were also obtained from female mice and similar trends were observed, except in female soleus muscle, where F_50_ was not different (Supplementary Figure 2), a peculiarity we discuss further in the discussion section.

**FIGURE 5:**
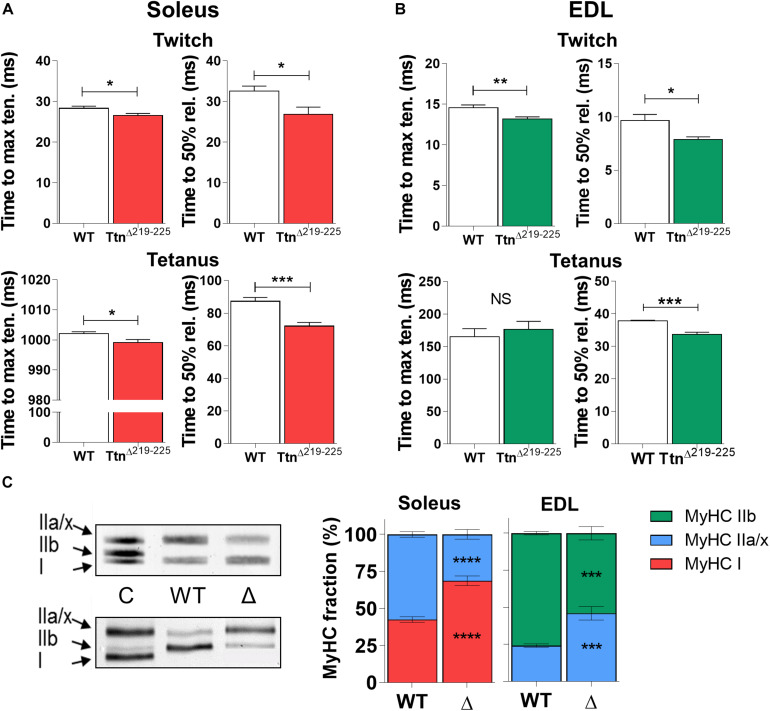
Kinetics of Ttn^Δ219–225^ muscles. Both soleus (**A**; *N* = 6–7) and EDL (**B**; *N* = 6–7) display increased contractile (left panel; time to maximum tension) and relaxation (right panel; time to 50% relaxation) kinetics during isometric twitch stimulations (top panels; 1 Hz). Only soleus shows similar increases in maximal tetanic stimulations (bottom panels; 150 Hz in soleus and 200 Hz in EDL) for time to maximal tension, a likely result from the fiber type switch seen in Figure C. Relaxation rate is also increased by 17% in the soleus and 11% in the EDL, suggesting the higher titin-based passive tension speeds up contractile and relaxation kinetics. **(C)** The kinetic changes coincided with a shift in myosin heavy chain (MyHC) type, showing a slow shift in both muscles of Ttn^Δ219–225^ mice (*N* = 8–10, males and females; representative MyHC gel of soleus; top, and EDL; bottom). C = wildtype tibialis/soleus mix used as running control to show successful separation of MyHC isoforms. *t*-test; *p < 0.05, **p < 0.01, ***p < 0.001 and NS, not significant.

The kinetics of force generation and force relaxation kinetics were studied during isometric twitch and maximal tetanic contractions. Soleus muscle (Figure 5A) showed reduced time to maximal tension during twitch contractions (28.3 ± 0.5 ms in WT; 26.6 ± 0.5 ms in Ttn^Δ219–225^, *p* = 0.035) and a minor reduction during maximal tetanic contractions (1002 ± 1 ms in WT to 999 ± 1 ms in Ttn^Δ219–225^, *p* = 0.026). Similarly, in EDL (Figure 5B) the time to maximal tension was reduced during twitch contractions (14.6 ± 0.3 in WT; 13.2 ± 0.3 in Ttn^Δ219–225^, *p* = 0.007). The time to maximal tension during tetanic contractions was not different (165 ± 13 in WT; 176 ± 13 in Ttn^Δ219–225^). The relaxation kinetics (time for tension to reduce by 50%) followed similar temporal shifts. Soleus muscle showed reduced time to half relaxation during twitch contractions (32.5 ± 0.5 ms in WT to 26.9 ± 0.5 ms in Ttn^Δ219–225^, *p* = 0.0228) and similarly during tetanic contractions (87.3 ± 2 ms in WT to 72.1 ± 2 ms in Ttn^Δ219–225^, *p* = 0.0007). In EDL, the time to half relaxation was reduced during twitch contraction (9.7 ± 0.6 in WT to 7.9 ± 0.2 in Ttn^Δ219–225^, *p* = 0.0109) and during tetanic contraction (37.8 ± 0.2 in WT to 33.6 ± 0.7 in Ttn^Δ219–225^, *p* = 0.0002).

To study whether the increase in F_50_ and the faster tension kinetics reflected a change in fiber type composition, the MyHC isoform composition of muscles was determined. Unexpectedly, soleus shifted from 42.6 ± 1.9% type I MyHC in WT to 68.7 ± 3.3% type I MyHC in Ttn^Δ219–225^ and EDL from 75.8 ± 1.1% type IIb MyHC in WT to 54.0 ± 4.5% type IIb MyHC in Ttn^Δ219–225^ (Figure 5C). The changes in fiber type correlate with the titin exon data (Supplementary Table 1), as slow-type muscle fibers have wider Z-discs and are expected therefore to express additional titin Z-repeats (Gregorio et al., 1998). However, the effect of this fiber type shift on F_50_ and tension kinetics is opposite of what was observed, i.e. a shift to slower fiber types lowers the F_50_ and slows tension kinetics (see section “Discussion”).

### Hypertrophy Sensitization of Ttn^Δ219–225^ Muscles

**FIGURE 6:**
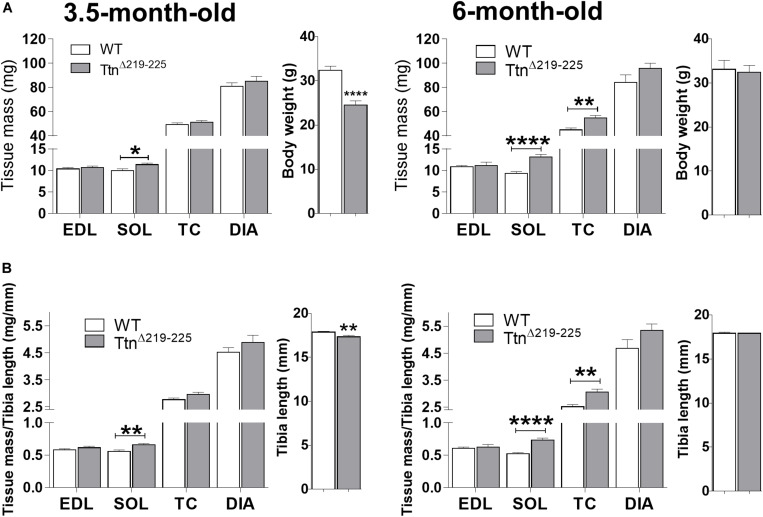
Muscle morphometric analysis. Absolute muscle weights of both 3.5-months (left) and 6-month (right) old male mice **(A,B)**. Ttn^Δ219–225^ mice display an early weight and skeletal system growth deficit [side panels **(A,B)**, respectively], compared to wildtype litter mates, which normalizes at 6 months of age (*N* = 11–14). Muscle weights are generally unaffected early on, except in the soleus (*p* < 0.01), with progressive increases in muscle mass as the mice age. *t*-test; *p < 0.05, **p < 0.01, ***p < 0.001, and ****p < 0.0001.

The effect of deleting exons 219–225 on skeletal muscle trophicity was also studied. Muscle mass (Figures 5A,B, left panel) was significantly higher in Ttn^Δ219–225^ soleus muscles of 3.5-month-old males (9.9 ± 0.4 mg in WT, 11.1 ± 0.4 mg in Ttn^Δ219–225^, *p* = 0.021), an increase that is absent in EDL muscle (10.4 ± 0.3 mg in WT to 10.6 ± 0.6 mg in Ttn^Δ219–225^). This higher muscle mass was also present in soleus muscle of 6-months-old males (9.3 ± 0.4 mg in WT to 13.2 ± 0.6 mg in Ttn^Δ219–225^, *p* > 0.0001), whereas EDL was not different between genotypes (10.8 ± 0.3 mg in WT to 11.2 ± 0.8 mg in Ttn^Δ219–225^). To further study if this difference in muscle mass was dependent on muscle type, we also determined the mass of two other predominantly fast-twitch muscles: tibialis cranialis (TC) and diaphragm (DIA) muscles. At 3.5-months-old, both TC (49.5 ± 1.3 mg in WT; 51.3 ± 1.3 mg in Ttn^Δ219–225^) and DIA (80.8 ± 3.0 mg in WT; 87.4 ± 3.7 mg in Ttn^Δ219–225^) muscles were not different. However, at 6-months-old (Figure 6, right panels) there was a significantly higher muscle mass in the TC (45.0 ± 1.6 mg in WT; 54.7 ± 2.2 mg in Ttn^Δ219–225^, *p* = 0.0023) and a trending increase in the DIA (84.2 ± 6.3 mg in WT; 95.6 ± 4.4 mg in Ttn^Δ219–225^, *p* = 0.1094). These findings suggest that the hypertrophy is not limited to predominantly slow-twitch muscles, and the mass increase in soleus (2-way ANOVA, *p* = 0.0123) and TC (2-way ANOVA, *p* = 0.0264) is progressive, hinting at a continuously active hypertrophy program. Ttn^Δ219–225^ male mice display early growth deficits, with a reduced body weight (Figure 6A, side panels) and a more diminutive skeleton size (as determined by tibia length measurements), which disappears at 6 months of age (Figure 6, right side panels). Female mice (Supplementary Figure 3), follow similar trends in muscle mass as males.

**FIGURE 7:**
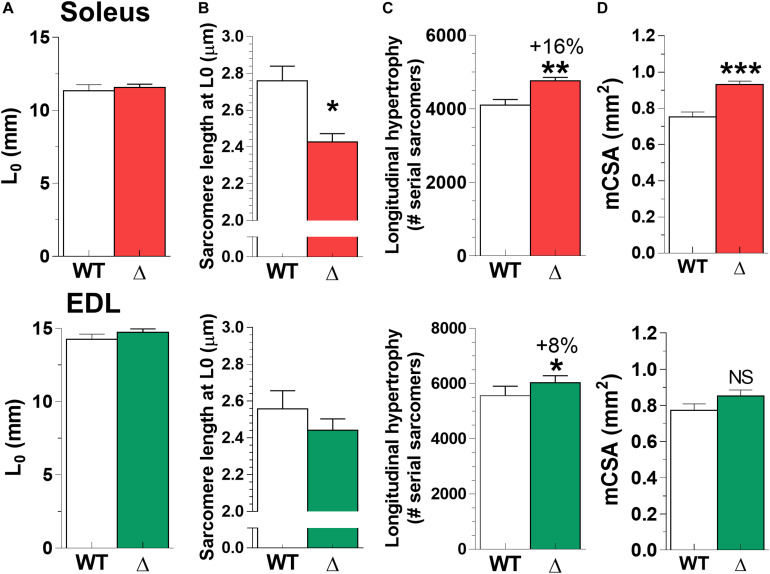
Longitudinal hypertrophy. To study whether longitudinal hypertrophy contributed to the mass increase (Figure 6), we used the L_0_ **(A)** and sarcomere length (SL; **B**), determined for Figure 4 and calculated the number of serial sarcomeres in both soleus (**C**; top panel) and EDL (bottom panel). Both Ttn^Δ219–225^ soleus and EDL have significantly more sarcomeres in series (*p < 0.05, **p < 0.01), with soleus having significantly (*p < 0.05) shorter sarcomeres at L_0_. **(D)** Muscle CSA was measured in passive muscles held at optimum length (L_0_). Soleus displayed cross-sectional hypertrophy, whereas CSA in EDL is unchanged (*N* = 6–7, males).

To study whether longitudinal hypertrophy contributed to the higher muscle mass in Ttn^Δ219–225^ muscles, we assessed the number of sarcomeres in series. We measured sarcomere lengths in formaldehyde fixed soleus and EDL set to their L_0_ lengths. Note that L_0_ was not different between WT and Ttn^Δ219–225^ muscles (Figure 7A). Sarcomere length was significantly shorter in soleus (Ttn^Δ219–225^ 2.42 ± 0.04 μm versus WT 2.76 ± 0.08 μm) and not different in EDL (and Ttn^Δ219–225^ 2.44 ± 0.06 μm versus WT 2.56 ± 0.10 μm; Figure 7B). Sarcomere length heterogeneity was comparable between wildtype soleus (coefficient of variation [CV] 7.82%) and EDL (CV 9.97%). There was less heterogeneity in the Ttn^Δ219–225^ (CV, SOL 4.76% and EDL 6.77%) muscles compared to WT. The sarcomere length data, taken together with the muscle length at L0, show that Ttn^Δ219–225^ muscles have significantly more sarcomeres in series: Ttn^Δ219–225^ soleus 4764 ± 95 versus WT soleus 4105 ± 154, EDL Ttn^Δ219–225^ 6031 ± 97 versus EDL WT 5572 ± 139. This represents a 16% increase in serial sarcomeres in the soleus and 8% in the EDL (Figure 7C; top and bottom panels, respectively).

In addition to longitudinal hypertrophy, we assessed muscle cross-sectional hypertrophy as a contributor to higher muscle mass. Soleus muscle showed significant increases in mCSA (Ttn^Δ219–225^ 0.93 ± 0.02 mm^2^ versus WT 0.75 ± 0.03 mm^2^, *p* < 0.001). In EDL no difference in mCSA was observed (Figure 7D). We also assessed gross morphology of both soleus and EDL muscle in H/E stained sections, and found muscles to display normal morphology, with increased fiber size in the soleus (Supplementary Figure 4). Thus, the 24% increase in mCSA of soleus, together with the increase in the number of serial sarcomeres, explains the higher muscle mass of soleus than of EDL.

**FIGURE 8:**
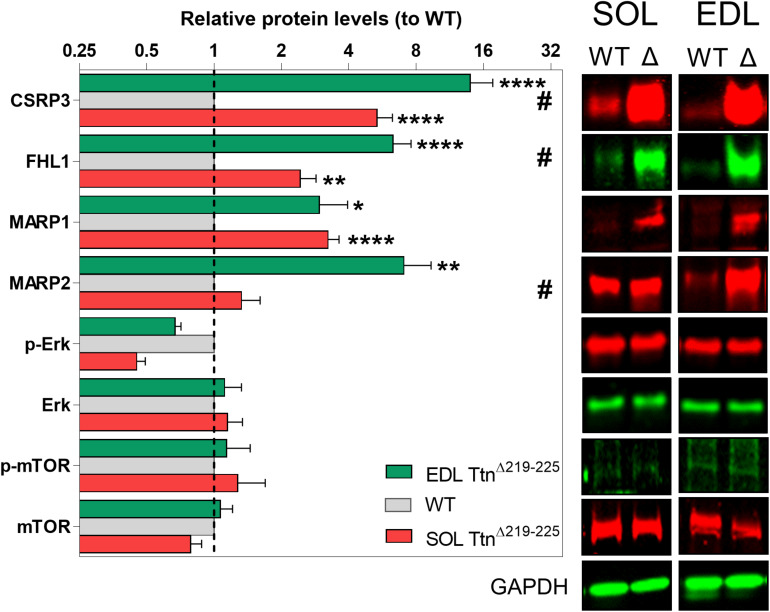
Western blots of various hypertrophy associated proteins. CSRP3, FHL1 and MARP2 show significant differences between soleus (red, lower bar) and EDL (green, upper bar) muscle, relative to wildtype muscles (WT; gray, middle bar), providing a possible link to the hypertrophy seen in the soleus, but not in the EDL muscle. ^#^*p* < 0.05 interaction factor 2-way ANOVA; *p < 0.05, **p < 0.01, ***p < 0.001, ****p < 0.0001 in repeated *t-*test.

To gain insight in the signaling mechanisms underlying hypertrophy of soleus muscle, Western blots were performed probing for titin-associated signaling proteins involved in trophic signaling [CSRP3, FHL1, MARP1, and MARP2] and proteins involved in protein synthesis pathways [Erk and mTOR] (Figure 8). Several proteins were upregulated (fold-increase compared to WT): CSRP3 (SOL 5.3 ± 0.1; EDL 13.9 ± 3.7), FHL1 (SOL 2.4 ± 0.4; EDL 6.3 ± 1.3), MARP1 (SOL 3.2 ± 0.4; EDL 2.9 ± 1.0), MARP2 (SOL 5.3 ± 0.1; EDL 13.9 ± 3.7), and Erk (SOL 0.6 ± 0.3; EDL 2.4 ± 0.8). Three of these proteins showed significant differences between soleus and EDL muscle: CSRP3, FHL1, and MARP2. Interestingly, these three proteins are all more strongly expressed in Ttn^Δ219–225^ EDL muscle, compared to Ttn^Δ219–225^ soleus muscle. As EDL does not show an increase in muscle mass, these proteins are unlikely to play a critical role in soleus hypertrophy.

**FIGURE 9:**
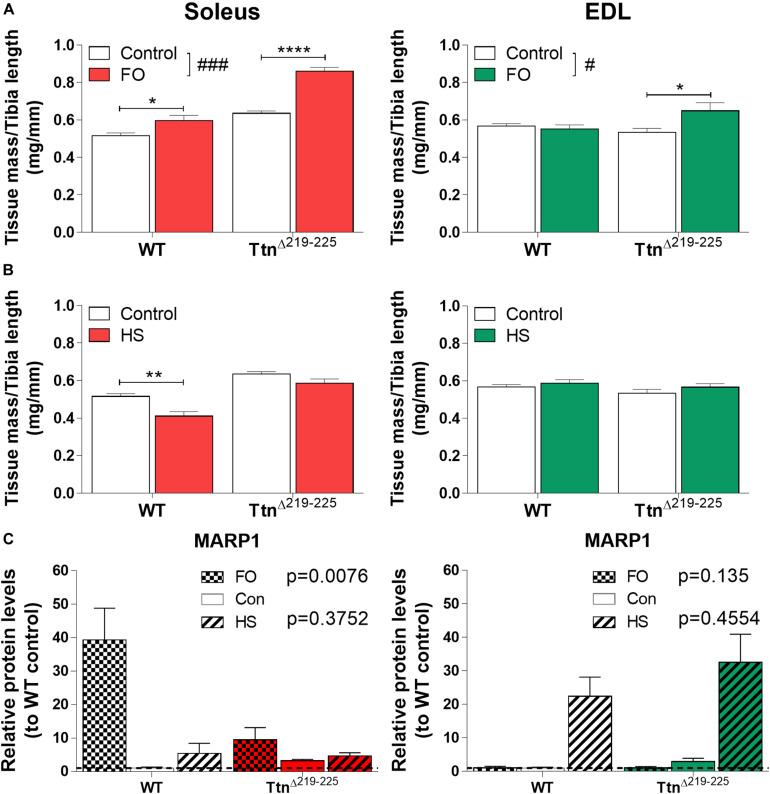
Response of Ttn^Δ219–225^ mice to muscle over- and unloading. Tissue mass normalized to tibia length, soleus (left) and EDL (right), with muscle functional overload due to ablation (**A**; FO;10 days) and unloading by hindlimb suspension (**B**; HS 10 days) (*N* = 6-8; 2-way ANOVA ^#^*p* < 0.05, ^###^*p* < 0.001, Tukey *post hoc* *p < 0.05, **p < 0.01, ****p < 0.0001). Western blot of MARP1 **(C)** showing opposing responses to FO and HS, implying a potential roll for MARP1 in muscle trophicity (*N* = 5-6, data represented as relative protein level compared to WT control; *p*-value from 2-way ANOVA on con. vs. FO and con. vs. HS, respectively).

To test whether Ttn^Δ219–225^ mice are more sensitive to muscle-load stimuli, we subjected mice to either functional overload (FO) of the soleus, by means of synergist ablation (surgical removal of gastrocnemius and plantaris muscle), or unloading by means of hindlimb suspension (HS). Following FO, soleus muscle mass was increased, with the increase being larger in Ttn^Δ219–225^ mice (WT 15.7% increase; Ttn^Δ219–225^ 35.2% increase). Interestingly, EDL muscles of Ttn^Δ219–225^ mice also developed hypertrophy (21.6% increase) (Figure 9A). Following HS, the soleus was atrophied in WT mice (mass 19.8% decrease), but this effect was attenuated in Ttn^Δ219–225^ (mass -7.9% decrease). There was no effect of HS in EDL of either WT or Ttn^Δ219–225^ mice (Figure 9B). This, together with the response of other muscles (Supplementary Figure 5), suggests that Ttn^Δ219–225^ mice are more sensitized to increase the muscle mass when exposed to a hypertrophy trigger, and more sensitized to resist muscle mass reductions when exposed to an atrophy trigger.

To explore a potential signaling mechanism underlying the hypertrophy sensitization, we probed several target proteins with Western blot. We found marked upregulation of CSRP3, FHL1 and MARP2 in Ttn^Δ219–225^ muscles (Supplementary Figure 6) in response to FO and/or HS. Only MARP1 had an opposing response between soleus and EDL (Figure 9C). Following FO, MARP1 was upregulated 39-fold in WT muscle, a response that was attenuated in Ttn^Δ219–225^ muscles (10-fold increase, *p* = 0.0076). HS only moderately affected MARP1 levels in soleus. EDL muscle showed the exact opposite response for MARP1, wherein FO showed no observable difference in MARP1 expression, but HS showed substantial upregulation of MARP in both WT and Ttn^Δ219–225^ muscles (22-fold increase in WT to 33-fold increase in Ttn^Δ219–225^). This opposing response of MARP1 in soleus compared to that in EDL muscle, following FO or HS, might imply a role during certain stages of hypertrophy development.

## Discussion

In this study, we characterized skeletal muscles in a mouse model with a small C-terminal deletion in titin’s PEVK segment. This deletion resulted in alternative splicing of titin’s I-band region, leading to the appearance of a second smaller isoform (N2A2), and an accompanying increase in the passive tension of skeletal muscle. The increase in stiffness of the Ttn^Δ219–225^ muscles coincided with faster kinetics of force generation and relaxation in slow-twitch soleus and fast-twitch EDL muscle, although the fraction of slow myosin heavy chain isoforms was increased. Ttn^Δ219–225^ mice presented with longitudinal and cross-sectional hypertrophy in soleus muscle, and a stronger response to hypertrophic stimuli.

### Passive Tension in the Ttn^Δ219–225^ Mouse Model

The Ttn^Δ219–225^ mouse model was originally generated to study the PEVK segment of the predominant titin isoform in the heart, N2B cardiac titin, and to resolve the function of the PEVK-segment in relation to biomechanics. In the heart, the Ttn^Δ219–225^ model displayed increased passive tension, suggesting that the PEVK segment is a major contributor to titin-based passive tension (Granzier et al., 2009). We expected to find little to no change in passive tension in skeletal muscle, due to the small size of the deletion relative to the long spring in skeletal muscle titin. In contrast, a large increase in passive tension was found. Calculations using a serially linked worm-like chain model indicated that excision of the seven exons only increases titin-based passive tension (17% at SL 3.0 μm; Figure 1B). However, a much larger passive tension increase was found (Figures 1C,D). This larger than expected passive tension increase is likely due to the appearance of an additional, smaller N2A isoform, referred to as N2A2 (Figure 2). However, the mechanical contribution of N2A2 is speculative, as we have not determined its incorporation in the sarcomere.

A similar N2A2 isoform was previously described in the Ttn^Δ30–38^ (proximal Ig domain deletion) (Buck et al., 2014) model. Soleus muscle from this mouse showed that ∼30% of total titin consisted of N2A2 and that titin-based passive tension was increased by ∼30% at SL 2.5 μm. Other models of titin I-band region deletion, such as the Ttn^Δ251–269^ (IA-junction deletion) (Granzier et al., 2014) and Ttn^Δ112–158^, do not show N2A2-like isoforms of titin in skeletal muscle. However, in the Ttn^Δ112–158^ model, most of the differentially spliced PEVK exons seen in the Ttn^Δ219–225^ and Ttn^Δ30–38^ models are deleted. For the Ttn^Δ251–269^ model it can be argued that that model should be considered an A-band region titin deletion model, due to its domain structure of Ig and fibronectin repeats. The only other reported titin I-band region deletion is a spontaneous deletion in the N2A and PEVK region. The corresponding mouse model, the muscular dystrophy with myositis (*mdm*; MGI: Ttn^*mdm*^) mouse model (Garvey et al., 2002), presents with only minor increases in passive tension. However, this mouse displays severe dystrophy, with increased ECM, potentially masking increased titin-based stiffness from an N2A2 isoform. Whether an N2A2 isoform is expressed in this model is unknown. An explanation for the additional splicing of titin’s I-band region in the Ttn^Δ219–225^ model might be related to the deletion of the phosphorylation sites that regulate titin stiffness. The deleted section in the Ttn^Δ219–225^ mouse contains 2 phosphorylation sites: S12742 (ex219) and S12884 (ex225) (van der Pijl et al., 2019). Both are phosphorylated by PKCα (Hidalgo et al., 2009) and CAMKIIδ (Hidalgo and Granzier, 2013). These sites increase titin-based stiffness when phosphorylated (Hudson et al., 2010). However, total phosphorylation levels were not different between WT and Ttn^Δ219–225^ muscles (Supplementary Figure 7), arguing against a role for this mechanism. Considering that titin has hundreds of potential post-translational modifications in its spring region, with only a few sites functionally described, it is difficult to assess their role in passive stiffness. Alternatively, there is the possibility that passive stiffness regulates Rbm20. Rbm20 is a known splice-factor of titin (Guo et al., 2012; Li et al., 2012; Murayama et al., 2018). Rbm20 signaling is potentially sensitive to the increased passive tension, resulting in increased expression or activation of Rbm20. Such a mechanism could also explain the N2A2 isoform in the Ttn^Δ30–38^ muscles.

### Titin and Active Force Development

There is substantial data supporting that titin-based stiffness modulates active force development by altering thin and thick-filament properties (Fukuda et al., 2008; Mateja et al., 2013; Elhamine et al., 2014; Li et al., 2016, 2019). Indeed, recent work in Ttn^Δ112–158^ muscle by Brynnel et al. (2018), showed that stiffer titin speeds the kinetics of active force generation, similar to the observations in the present study (relaxation kinetics were not determined in Ttn^Δ112–158^ muscle). Recently, advances have been made in the understanding of the mechanisms by which titin stiffness modulates contraction kinetics. Stiffer titin molecules put more strain on the thick filaments (Irving et al., 2011). The increased strain promotes the thick filaments “ON”-state, thereby accelerating force kinetics (Fusi et al., 2016; Piazzesi et al., 2018). We propose that the increased force generation and relaxation kinetics underlie the increased F_50_ stimulation frequency (Figure 4B, inserts). Due to the increased kinetics, force summation occurs at higher stimulation frequencies. Apparently, this effect is quite pronounced, as it outweighs the reduced force kinetics due to the fast-to-slow fiber type shift. The increased F_50_ was less evident in female muscles (Supplementary Figure 2). However, muscles of female Ttn^Δ219–225^ mice also present with an increased proportion of type 1 fibers (Figure 5C), which would decrease the F_50_ in soleus. Thus, this suggests that the F_50_ in Ttn^Δ219–225^ females is actually increased. Perhaps females do not switch fiber type to the same extent as the males, something that was not tested in this manuscript, resulting in a less pronounced increase in F_50_ compared to that of male Ttn^Δ219–225^ mice.

These findings prompted us to measure the force kinetics in both WT and Ttn^Δ219–225^ muscles set at the same baseline passive tension (matched preload, Supplementary Figure 8). Interestingly, at matched preload the contraction kinetics are slower in Ttn^Δ219–225^ muscle compared to WT, in line with the slow fiber type switch. This finding supports that titin-based passive tension modulates the force generation and relaxation kinetics. However, it should be noted that matched pre-loads were achieved at different sarcomere length and we cannot rule out that this affected the results via a passive tension-independent mechanism.

### Trophic Sensitization of Slow Twitch Muscle

The increased absolute force production in (slow-twitch) soleus muscle of the Ttn^Δ219–225^ model is in part due to cross-sectional hypertrophy (Figure 7 and Supplementary Figure 4). The cross-sectional hypertrophy, combined with the longitudinal hypertrophy, accounts for the observed increased muscle mass in the Ttn^Δ219–225^ model. The hypertrophy is not apparent in (fast-twitch) EDL muscle, suggesting that it is muscle specific. The muscles that do hypertrophy, show progressive growth (Figure 6). Tibialis muscle (TC) has significant increases in mass at 6 months, with a similar trend for the diaphragm. TC and diaphragm are fast-twitch muscles, suggesting that the hypertrophy is not fiber type specific and more likely correlates with the passive characteristics of the muscle. Perhaps the hypertrophy can be attributed to the operating sarcomere length range of the muscles. EDL muscle operates at a smaller sarcomere length range compared to soleus (Burkholder and Lieber, 2001; Brynnel et al., 2018). This smaller range reduces the amplitude of the passive tension change. Previous work suggests that large fluctuations in passive tension induce both cross sectional and longitudinal hypertrophy, with increased titin stiffness amplifying the effect (van der Pijl et al., 2018). Thus, the smaller sarcomere length range in EDL might blunt the stimulus for longitudinal and cross-sectional adaptations in Ttn^Δ219–225^ muscle, resulting in less change in muscle mass.

Longitudinal hypertrophy is expected to normalize the passive stiffness of muscles at their operating sarcomere length range. This notion is supported by recent work on the Ttn^Δ112–158^ model, which showed normalization of passive tension due to addition of sarcomeres in series (Brynnel et al., 2018). Thus, titin-based passive stiffness controls the number of sarcomeres in series, probably to maintain passive tension within a desired range. To determine how this addition in serial sarcomeres affects muscle stiffness, we calculated the average passive tension (based on total passive tension; Figures 1C,D) in a sarcomere length range of 2.3–3.0 μm, including an addition of 16% serial sarcomeres in soleus and 8% in EDL (calculated as percentage decrease in stiffness; Supplementary Figure 9; middle panels). This addition of sarcomeres shifts the sarcomere length range in the Ttn^Δ219–225^ muscles to 1.93–2.52 μm in soleus and to 2.12–2.76 μm in EDL (Supplementary Figure 9; bottom panels, respectively). Consequently, this results in a decrease in passive stiffness in soleus (Supplementary Figure 9A; middle panel) and a similar trend in EDL (Supplementary Figure 9B; middle panel). Thus, the longitudinal hypertrophy might be a compensatory mechanism to adjust for the increase in passive stiffness.

To gain insight into the hypertrophy signaling mechanisms at play, we probed both soleus and EDL muscles for titin-binding proteins and protein synthesis regulators (Figure 8). Although we did identify several upregulated proteins, they appear to be more related to cellular stress and less so to trophicity. Neither of the probed protein synthesis pathways, mTOR and Erk, showed convincing levels of activity, as determined by phosphorylation of mTOR S2181 (auto-phosphorylation site for kinase activity) and Erk Thr202/Tyr204 (dual phosphorylation for downstream effector regulation). Likely the signaling processes in the Ttn^Δ219–225^ mouse are only moderately active, which makes it challenging to deduce the underlying mechanisms.

Finally, a striking finding was that both EDL and soleus from Ttn^Δ219–225^ mice showed a sensitization to hypertrophic stimuli (Figure 9). Repeating the Western blot panel, we found MARP1 to stand out as a potential regulator of hypertrophy. MARP1 was oppositely regulated, during over- and unloading, between soleus and EDL muscle. MARP1 in soleus was sensitive to functional overload, but was significantly less responsive in the Ttn^Δ219–225^ mouse. In EDL, hindlimb suspension rather than functional overload was the main activator of MARP1, independent of genotype. Considering the strong hypertrophy response of soleus muscle in the Ttn^Δ219–225^ mice to functional overload (Figure 9A), MARP1 might be a negative regulator of hypertrophy. Such a mechanism, however, is speculative. The Ttn^Δ219–225^ muscles are sensitized to hypertrophy, i.e. both soleus and EDL responded stronger to muscle overload, and showed a blunted response to unloading (Figure 9B). The mechanisms underlying the sensitization are unclear. We speculate that the C-terminal portion of the PEVK segment houses a signaling complex that normally suppresses hypertrophy but this and other possibilities require future study.

## Conclusion

We hypothesized that the Ttn^Δ219–225^ model would display minimally altered passive tension and remodeling in skeletal muscle. Surprisingly, the deletion of these seven exons increased passive tension several times more than predicted, likely at least in part due to unanticipated alternative splicing of the titin transcript and resulting in stiffer titin isoforms. Consequently, Ttn^Δ219–225^ muscle shows extensive remodeling, including longitudinal and cross-sectional hypertrophy, and changes in muscle contractility. The results support the notion that titin-based stiffness controls the number of serial sarcomeres.

## Data Availability Statement

All datasets generated for this study are included in the article/Supplementary Material.

## Ethics Statement

This study was performed in accordance with the Guide for the Care and Use of Laboratory Animals of the National Institutes of Health. All animal experiments were performed according to approved institutional animal care and use committee (IACUC) protocols (#09-095 & #13-488) of the University of Arizona.

## Author Contributions

MG, CO, and HG designed the research studies. BH, RP, TG-N, FL, AK, JS, and CC conducted the experiments and acquired the data. RP, BH, FL, JS, and CC analyzed the data. RP, MG, HG, and CO wrote the manuscript.

## Conflict of Interest

The authors declare that the research was conducted in the absence of any commercial or financial relationships that could be construed as a potential conflict of interest.

## Supplementary Material

FIGURE S1 Simulated stiffness of N2A2 titin. Simulated stiffness of wildtype (WT; black line) and Ttn^Δ219–225^ (red line) molecules. The green line shows stiffness in case 100% of the molecules consist of N2A2. The blue line shows stiffness in case 20% of the molecules consists of N2A2.

FIGURE S2 Active muscle properties of female Ttn^Δ219–225^ mice. **(A,B)** active tension of soleus (WT, black line; Ttn^Δ219–225^ red line) and EDL (WT, black line; Ttn^Δ219–225^ green line), respectively. Curve fitting revealed a significant increase in tetanic tension (^****^*p* < 0.0001) for the soleus and decrease (^****^*p* < 0.0001) in the EDL, comparable to what was seen in males (Figure 4B). Insert depict the frequency that produces 50% of the maximum active tension (F_50_). Soleus is not right shifted at submaximal activation, while EDL is similarly right shifted as seen in the males (Figure 4B). **(C)** Muscle CSA was measured in passive muscles held at optimum length. Soleus (top) displayed cross-sectional hypertrophy, whereas EDL (bottom) is unchanged (*N* = 7–8).

FIGURE S3 Muscle morphometric analysis of female mice. Absolute muscle weights **(A,B)** of both 3.5-months (left) and 6-month (right) old mice. Contrary to males, females Ttn^Δ219–225^ mice display a late weight deficit (inserts B; *p* = 0.0546) compared to wildtype litter mates. Muscle weights are generally unaffected early on, except in the soleus (*p* < 0.01), with progressive increases in muscle mass as the mice age (*N* = 6–11).

FIGURE S4 Muscle morphometry of soleus and EDL muscle. Representative images of hematoxylin and eosin stained cryosections of wildtype and Ttn^Δ219–225^ soleus and EDL muscle (*N* = 4, for each muscle). Gross morphology is preserved in Ttn^Δ219–225^ mice. Ttn^Δ219–225^ soleus shows larger fibers, consistent with hypertrophy, and EDL shows smaller fibers, consistent with fiber type switching to more 2a/x fibers (see Figure 5C).

FIGURE S5 Response of Ttn^Δ219–225^ mice to muscle over and unloading. Tissue mass normalized to tibia length, with muscle functional overload represented by ablation (FO; left; 10 days) and unloading by hind limb suspension (HS; right; 10 days). **(A)** Tibialis cranialis (TC). **(B)** Quadriceps (Quad). **(C)** Left ventricle (LV). **(D)** Right ventricle (RV), (*N* = 6–8, males; 2-way ANOVA NS; Sidak *post hoc* ^∗∗^*p* < 0.01, ^****^*p* < 0.00001).

FIGURE S6 Hypertrophy signaling of Ttn^Δ219–225^ mice in response to muscle over- and unloading. Muscle functional overload represented by ablation (FO) and unloading by hindlimb suspension (HS). Soleus in red and EDL in green, data represented as relative protein level compared to WT control (*N* = 5–6; 2-way ANOVA).

FIGURE S7 Titin phosphorylation. Relative ProQ Diamond to Sypro Ruby red signal of titin, in both soleus muscle (left) and EDL muscle (right) (*N* = 6 for all groups).

FIGURE S8 Effect of preload in Ttn^Δ219–225^ mice. Relative force plotted against time to 50% activation, max activation and 50% relaxation. Twitch activation (stimulation at 1 Hz) measured at optimum length (L0; **(A)** soleus, **(B)** EDL; *N* = 16–17, males and females) show that both muscles have faster relaxation kinetics (# RM 2-way ANOVA; ^∗^Holm Sidak *post hoc*). Since both muscles show a slow fiber switch (Figure 5C), we tested if stiffness affects relaxation time. Muscle set to a length where they reached 85mg preload, matched stiffness (MS) and measured with the same twitch protocol. Soleus (**C**, *N* = 7–8) shows a right shift, consistent with the slow fiber switch, however, EDL (**D**, *N* = 7–13) remains faster in 50% relaxation time, suggesting titin affects relaxation kinetics.

FIGURE S9 Effect of additional serial sarcomeres on passive stiffness. To determine the effect of additional serial sarcomeres in both soleus (**A**; top panel) and EDL (**B**; top panel) on passive tension, we calculated the effect of adding 16% (Soleus) and 8% (EDL) additional sarcomeres as tension reduction (total passive tension, Figure 1), within a SL range of 2.3-3.0 μm (middle panels) and determined stiffness. The additional sarcomeres in the Ttn^Δ219–225^ soleus normalize the stiffness, in theory, by shifting sarcomere working range, with a similar trend in EDL (bottom panels).

TABLE S1 Titin exon array. Mouse titin exon expression data of soleus and EDL muscle. Exon nomenclature according to the human titin sequence retreived from Ensmbl ENSG00000155657.

TABLE S2 Antibodies used in this study.
