# Supplementary figures and images for: Deleting Titin’s C-Terminal PEVK Exons Increases Passive Stiffness, Alters Splicing, and Induces Cross-Sectional and Longitudinal Hypertrophy in Skeletal Muscle
Source: Front Physiol. 2020 May 29;11:494. doi: 10.3389/fphys.2020.00494 (PMC7274174; doi:10.3389/fphys.2020.00494)

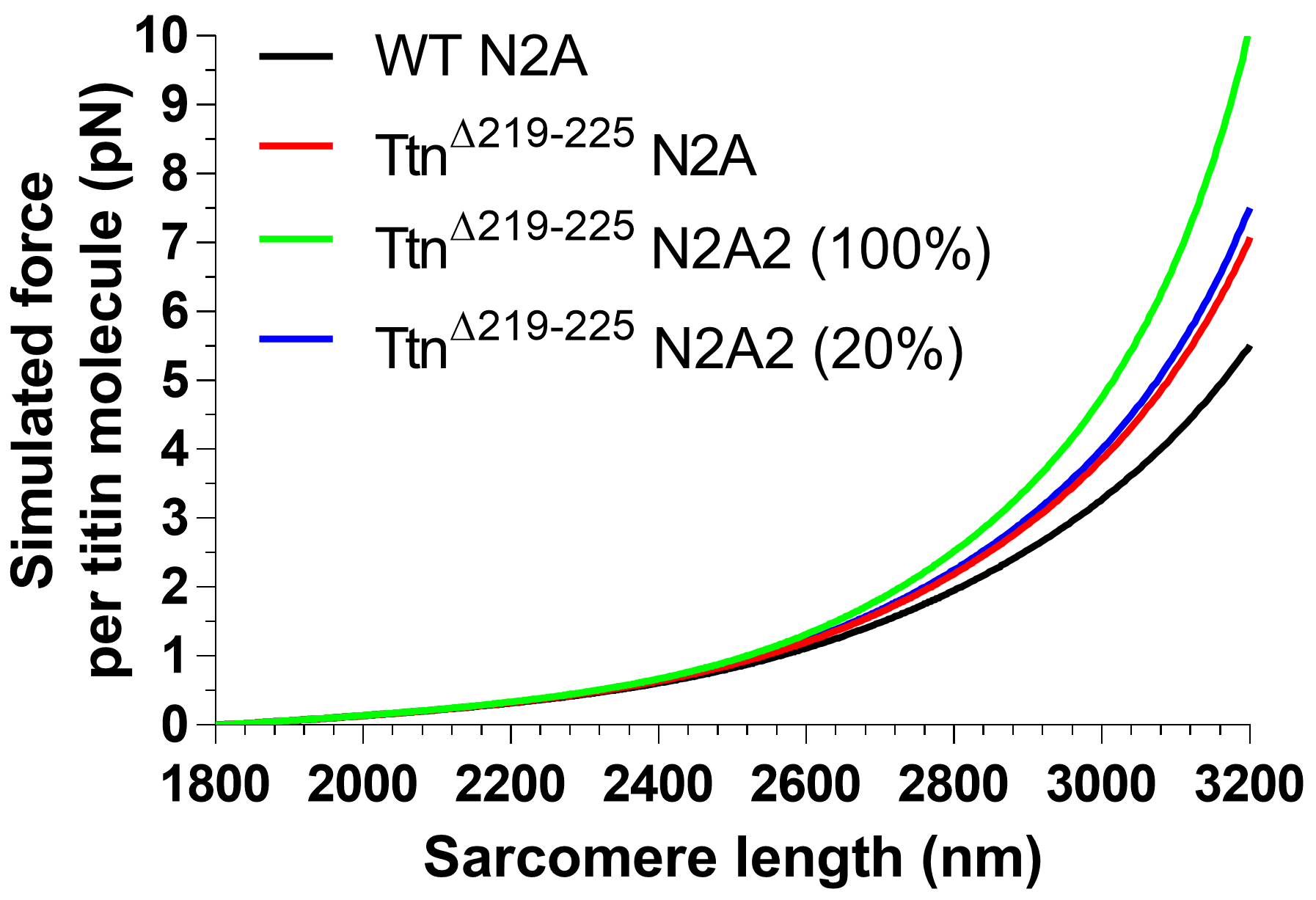

Supplement: FIGURE S1 — Simulated stiffness of N2A2 titin. Simulated stiffness of wildtype (WT; black line) and TtnΔ219–225 (red line) molecules. The green line shows stiffness in case 100% of the molecules consist of N2A2. The blue line shows stiffness in case 20% of the molecules consists of N2A2. [file Image_1.TIF]

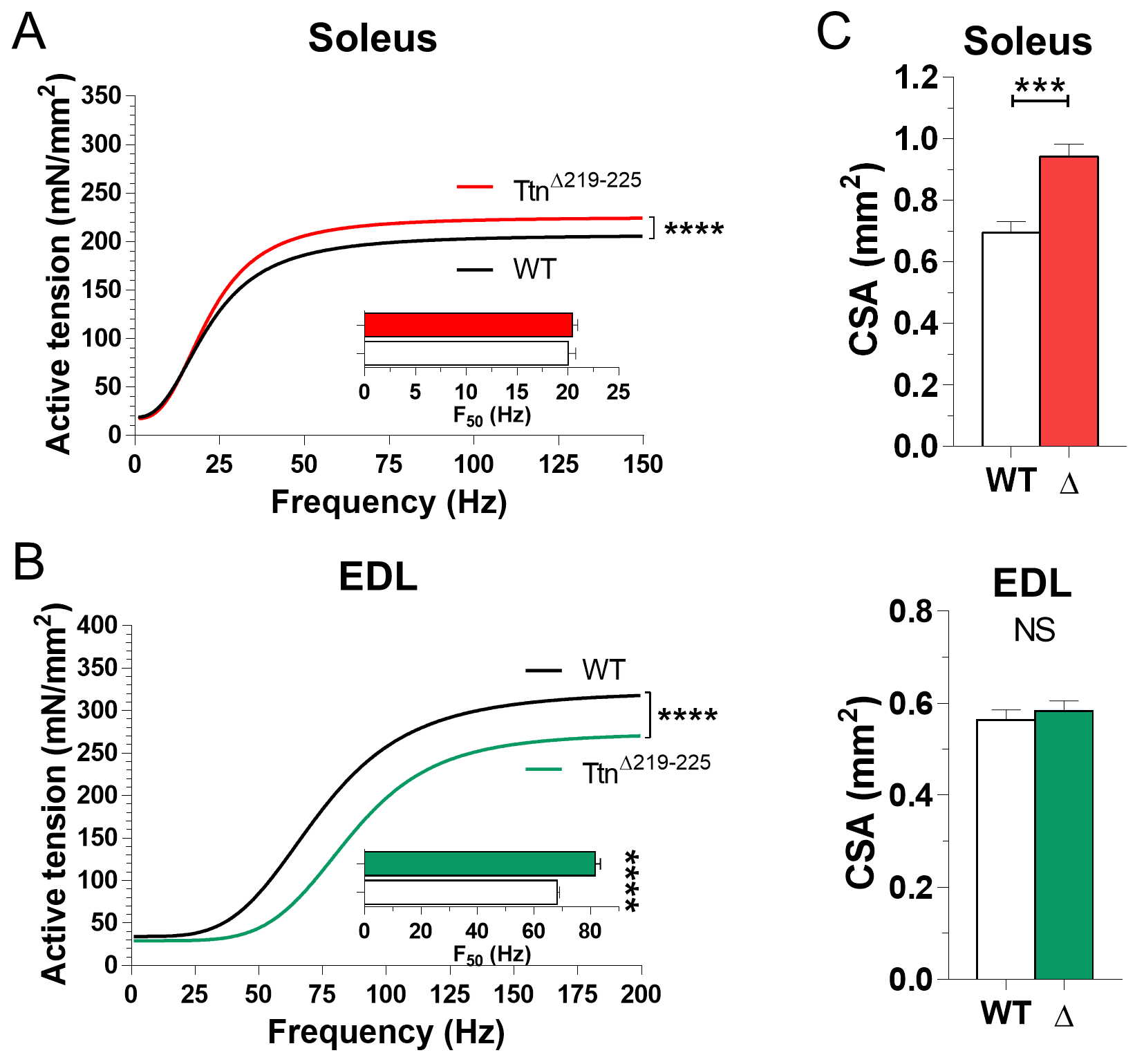

Supplement: FIGURE S2 — Active muscle properties of female TtnΔ219–225 mice. (A,B) active tension of soleus (WT, black line; TtnΔ219–225 red line) and EDL (WT, black line; TtnΔ219–225 green line), respectively. Curve fitting revealed a significant increase in tetanic tension (****p < 0.0001) for the soleus and decrease (****p < 0.0001) in the EDL, comparable to what was seen in males (Figure 4B). Insert depict the frequency that produces 50% of the maximum active tension (F50). Soleus is not right shifted at submaximal activation, while EDL is similarly right shifted as seen in the males (Figure 4B). (C) Muscle CSA was measured in passive muscles held at optimum length. Soleus (top) displayed cross-sectional hypertrophy, whereas EDL (bottom) is unchanged (N = 7–8). [file Image_2.TIF]

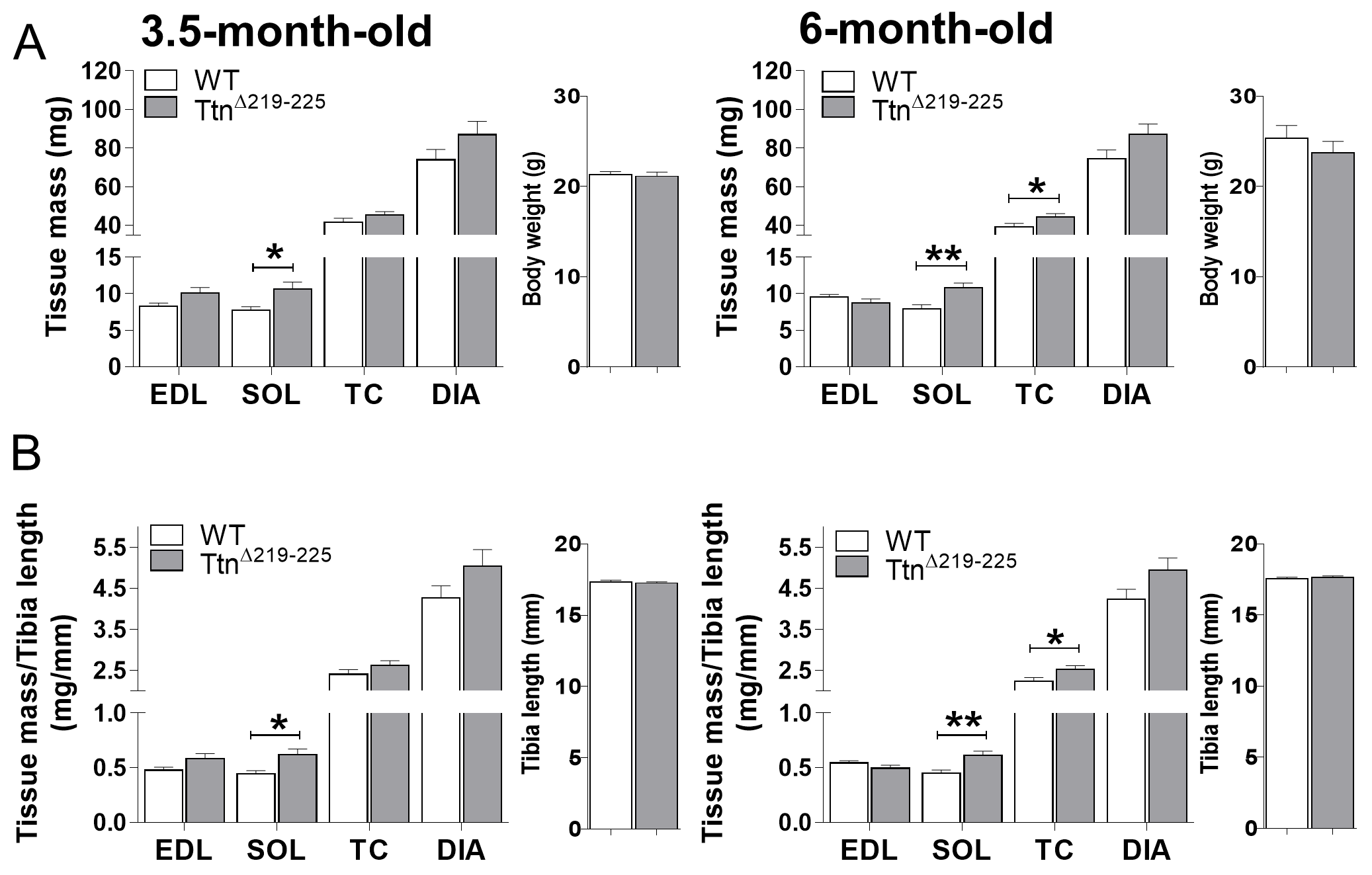

Supplement: FIGURE S3 — Muscle morphometric analysis of female mice. Absolute muscle weights (A,B) of both 3.5-months (left) and 6-month (right) old mice. Contrary to males, females TtnΔ219–225 mice display a late weight deficit (inserts B; p = 0.0546) compared to wildtype litter mates. Muscle weights are generally unaffected early on, except in the soleus (p < 0.01), with progressive increases in muscle mass as the mice age (N = 6–11). [file Image_3.TIF]

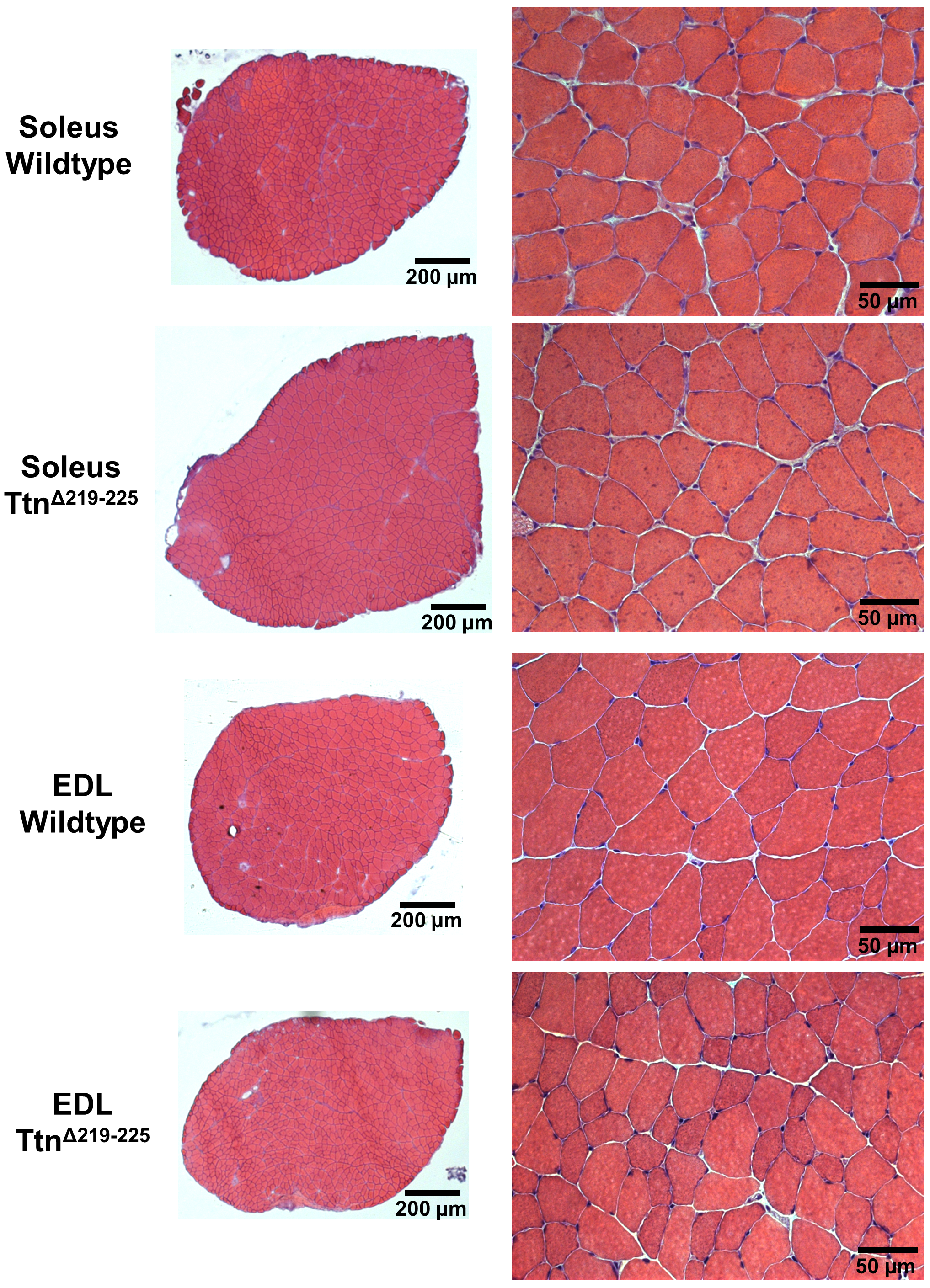

Supplement: FIGURE S4 — Muscle morphometry of soleus and EDL muscle. Representative images of hematoxylin and eosin stained cryosections of wildtype and TtnΔ219–225 soleus and EDL muscle (N = 4, for each muscle). Gross morphology is preserved in TtnΔ219–225 mice. TtnΔ219–225 soleus shows larger fibers, consistent with hypertrophy, and EDL shows smaller fibers, consistent with fiber type switching to more 2a/x fibers (see Figure 5C). [file Image_4.TIF]

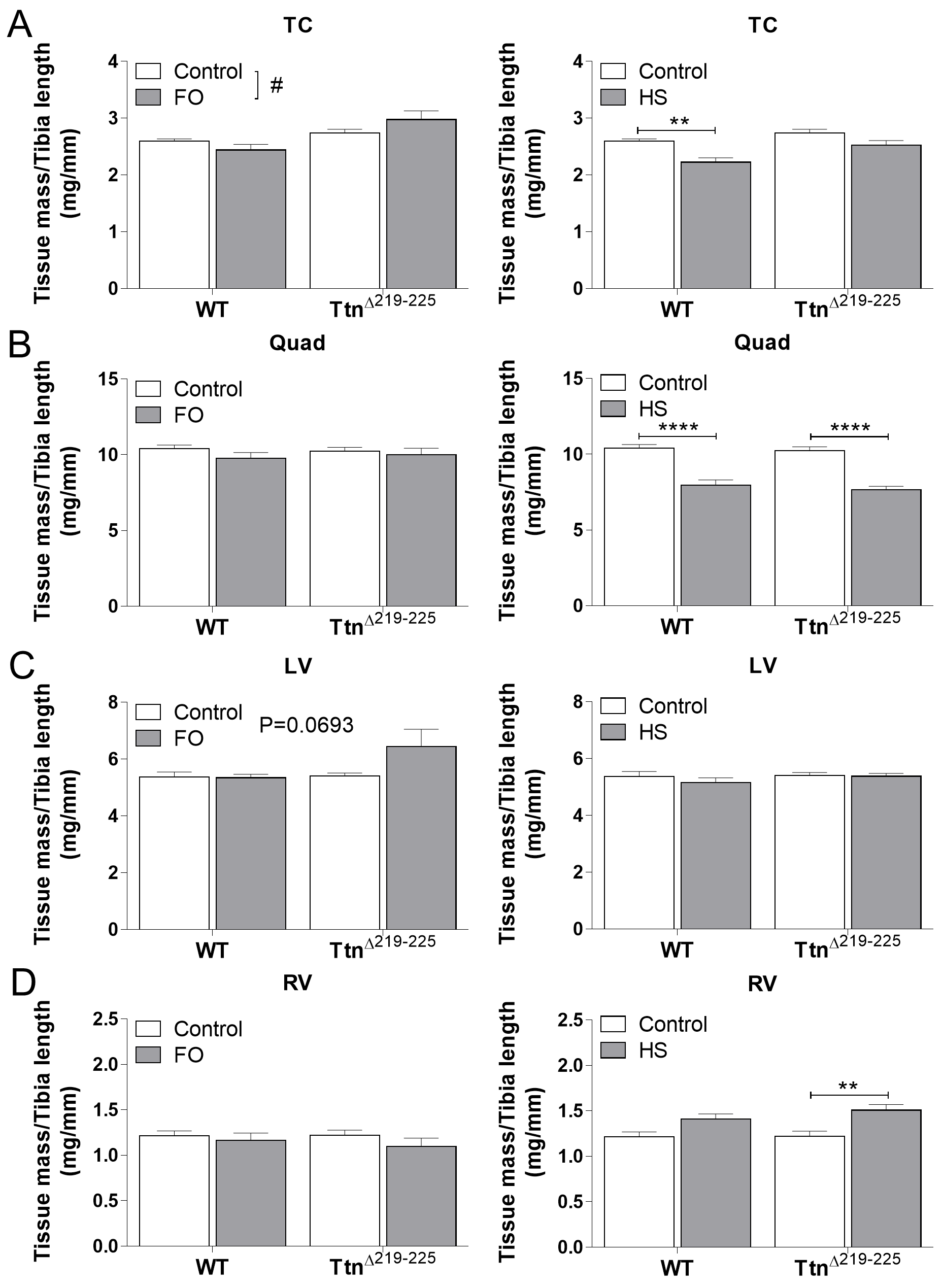

Supplement: FIGURE S5 — Response of TtnΔ219–225 mice to muscle over and unloading. Tissue mass normalized to tibia length, with muscle functional overload represented by ablation (FO; left; 10 days) and unloading by hind limb suspension (HS; right; 10 days). (A) Tibialis cranialis (TC). (B) Quadriceps (Quad). (C) Left ventricle (LV). (D) Right ventricle (RV), (N = 6–8, males; 2-way ANOVA NS; Sidak post hoc ∗∗p < 0.01, ****p < 0.00001). [file Image_5.TIF]

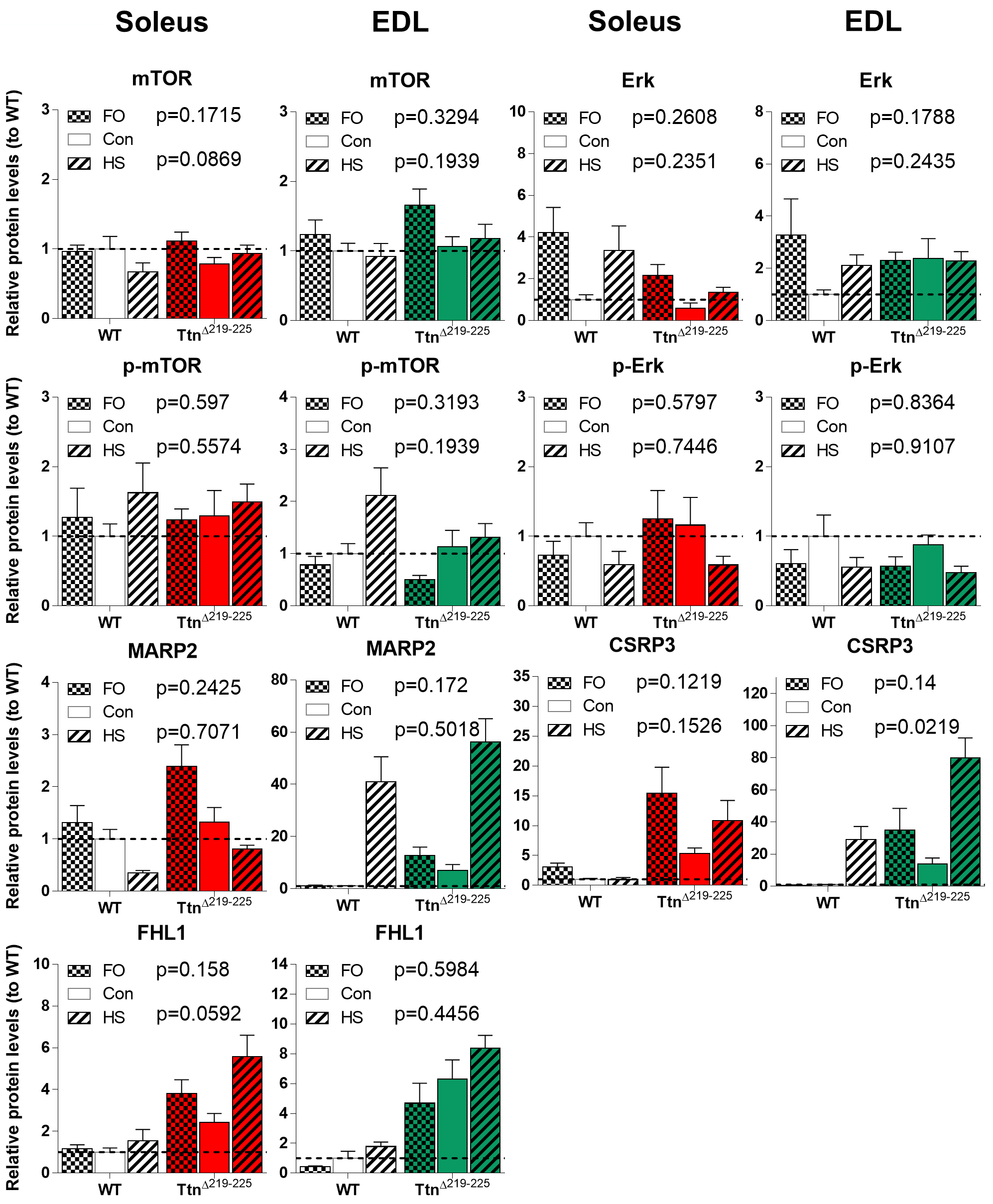

Supplement: FIGURE S6 — Hypertrophy signaling of TtnΔ219–225 mice in response to muscle over- and unloading. Muscle functional overload represented by ablation (FO) and unloading by hindlimb suspension (HS). Soleus in red and EDL in green, data represented as relative protein level compared to WT control (N = 5–6; 2-way ANOVA). [file Image_6.TIF]

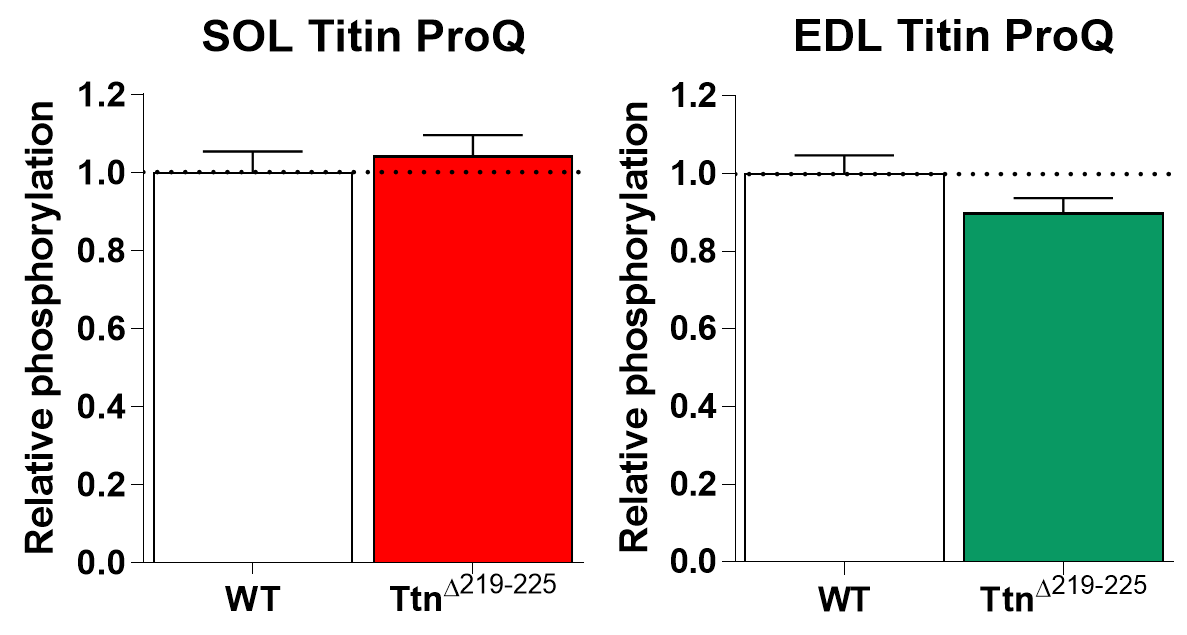

Supplement: FIGURE S7 — Titin phosphorylation. Relative ProQ Diamond to Sypro Ruby red signal of titin, in both soleus muscle (left) and EDL muscle (right) (N = 6 for all groups). [file Image_7.TIF]

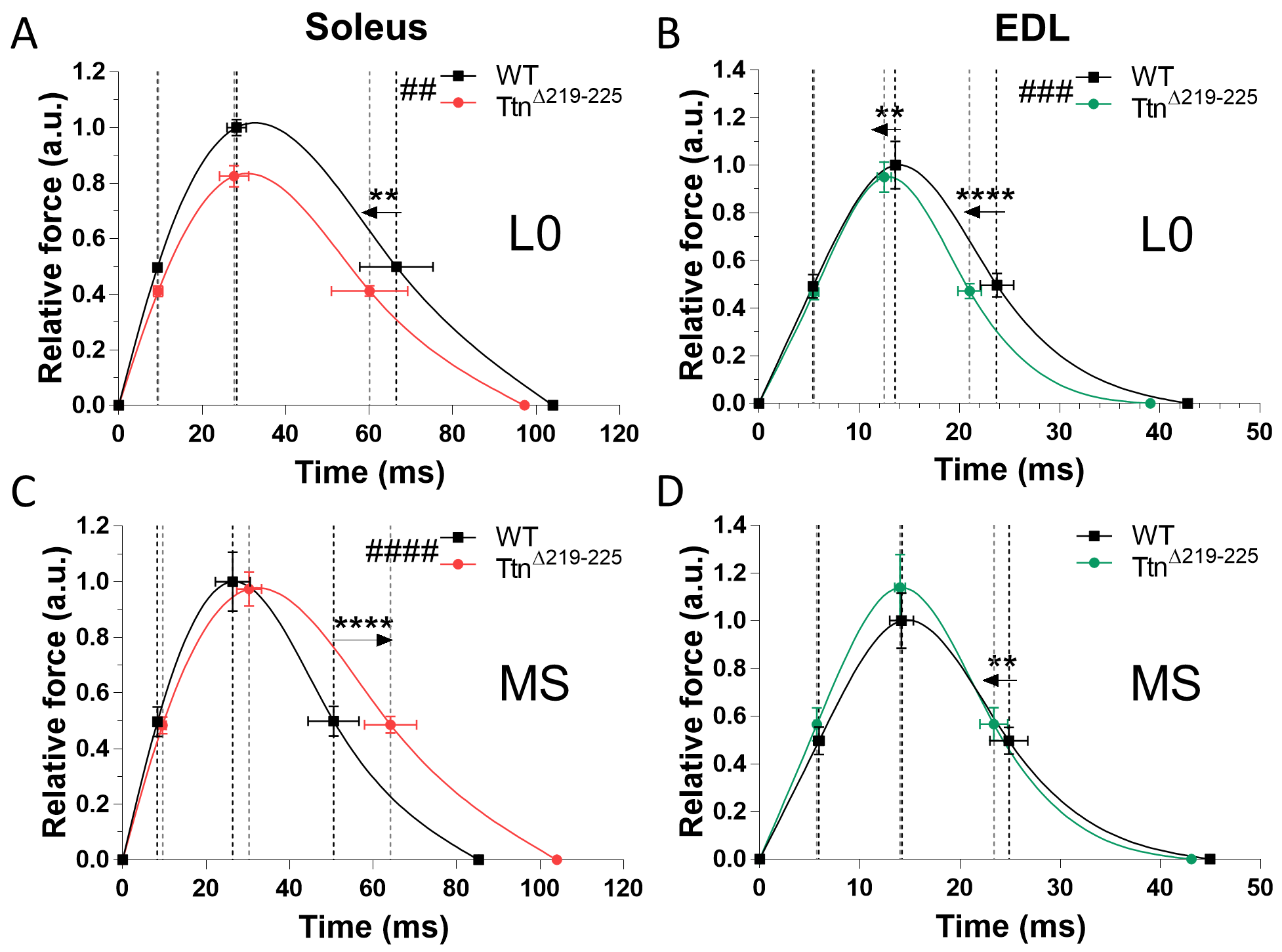

Supplement: FIGURE S8 — Effect of preload in TtnΔ219–225 mice. Relative force plotted against time to 50% activation, max activation and 50% relaxation. Twitch activation (stimulation at 1 Hz) measured at optimum length (L0; (A) soleus, (B) EDL; N = 16–17, males and females) show that both muscles have faster relaxation kinetics (# RM 2-way ANOVA; ∗Holm Sidak post hoc). Since both muscles show a slow fiber switch (Figure 5C), we tested if stiffness affects relaxation time. Muscle set to a length where they reached 85mg preload, matched stiffness (MS) and measured with the same twitch protocol. Soleus (C, N = 7–8) shows a right shift, consistent with the slow fiber switch, however, EDL (D, N = 7–13) remains faster in 50% relaxation time, suggesting titin affects relaxation kinetics. [file Image_8.TIF]

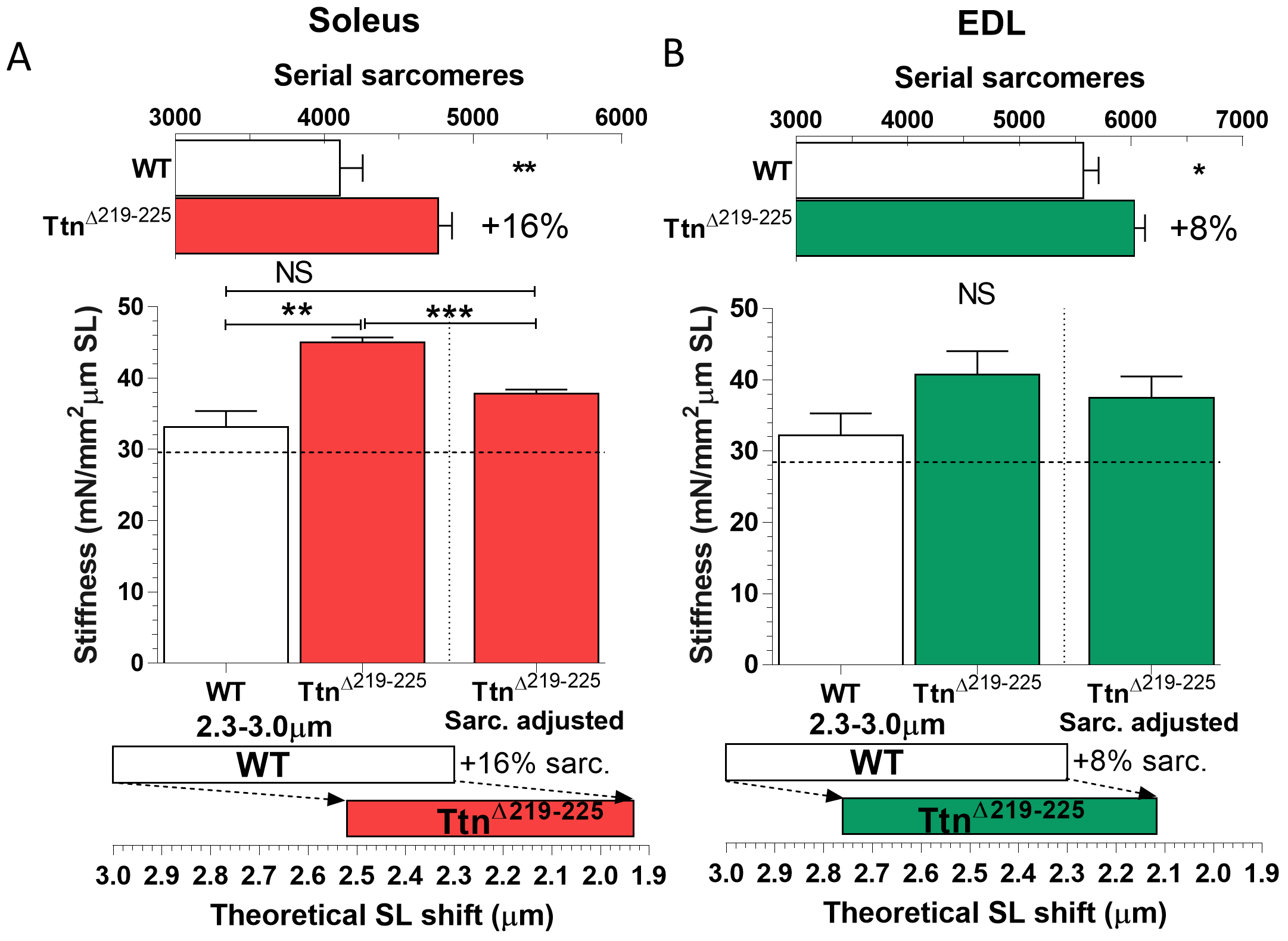

Supplement: FIGURE S9 — Effect of additional serial sarcomeres on passive stiffness. To determine the effect of additional serial sarcomeres in both soleus (A; top panel) and EDL (B; top panel) on passive tension, we calculated the effect of adding 16% (Soleus) and 8% (EDL) additional sarcomeres as tension reduction (total passive tension, Figure 1), within a SL range of 2.3-3.0 μm (middle panels) and determined stiffness. The additional sarcomeres in the TtnΔ219–225 soleus normalize the stiffness, in theory, by shifting sarcomere working range, with a similar trend in EDL (bottom panels). [file Image_9.TIF]
