# Supplementary material for: Deleting Titin’s C-Terminal PEVK Exons Increases Passive Stiffness, Alters Splicing, and Induces Cross-Sectional and Longitudinal Hypertrophy in Skeletal Muscle
Source: Front Physiol. 2020 May 29;11:494. doi: 10.3389/fphys.2020.00494 (PMC7274174; doi:10.3389/fphys.2020.00494)
Supplement: TABLE S2 — Antibodies used in this study. [file Table_2.docx]

| **Antibodies** | **Source** | **Clonality (clone)** | **Dilution** | **Company** |
| --- | --- | --- | --- | --- |
| Rbm20 | Rabbit | polyclonal | 1:500 | Myomedix |
| CSRP3/MLP | Rabbit | polyclonal | 1:2500 | Myomedix |
| MARP1 | Rabbit | polyclonal | 1:500 | Myomedix |
| MARP2 | Rabbit | polyclonal | 1:2000 | Myomedix |
| Ttn C-term (m8/m9) | Rabbit | polyclonal | 1:1000 | Myomedix |
| Ttn N-term | Mouse | monoclonal (M06) | 1:500 | Abnova |
| FHL1 | Mouse | monoclonal (ba535K.18) | 1:500 | Abcam |
| p44/42 MApK (Erk) | Rabbit | monoclonal (L34F12) | 1:250 | Cell Signaling |
| Phospho p44/42 MApK (Thr202/Tyr204) | Rabbit | polyclonal | 1:500 | Cell Signaling |
| Gapdh | Rabbit | monoclonal (14C10) | 1:5000 | Cell Signaling |
| mTOR | Mouse | monoclonal (7C10) | 1:750 | Cell Signalling |
| S2481 p-mTOR | Rabbit | polyclonal | 1:500 | Santa Cruz |
| Gapdh | Mouse | monoclonal (GA1R) | 1:3000 | Pierce |
| CF680 Goat anti-Rabbit IgG | Goat | polyclonal | 1:20.000 | Biotium |
| CF790 Goat anti-Mouse IgG | Goat | polyclonal | 1:20.000 | Biotium |

Table 2. Antibodies used in this study
