# Supplementary material for: Deleting Titin’s C-Terminal PEVK Exons Increases Passive Stiffness, Alters Splicing, and Induces Cross-Sectional and Longitudinal Hypertrophy in Skeletal Muscle
Source: Front Physiol. 2020 May 29;11:494. doi: 10.3389/fphys.2020.00494 (PMC7274174; doi:10.3389/fphys.2020.00494)
Supplement: Supplementary file 12 [file Table_3.docx]

**Supplemental methods**

**Titin phosphorylation.** Vertical SDS–agarose electrophoresis (SDS-AGE) was performed as described in the main manuscript. Gels were stained with ProQ diamond (Invitrogen), followed by Sypro ruby red (invitrogen) and scanned using a GBOX (Syngene; United Kingdom). Fluorescent intensity was measured using Image J (NIH, USA) and total phosphorylation was determined by dividing the ProQ diamond signal over the Sypro ruby red signal.

**Histology.** Freshly dissected soleus and EDL muscles, from both wildtype and Ttn^Δ219-225^ mice, were imbedded in OCT compound by freezing, through isopentane, in liquid nitrogen. 6 µm section were cut on a Microm HM 550 cryostat (Thermo Scientific; USA) and collected on superfrost plus slides. Sections were stained with hematoxylin (according to Harris) and eosin (1%, aqueous) following standard protocols. Images were captured using an AxioCam MRc microscope, with Axiovision software (Zeiss; Oberkochen, Germany), at ×50 magnification for complete cross sections and ×400 for detail images. Images were calibrated using a 0.01 mm stage micrometre (Edmund Optics; Barrington, USA).

**Muscle contractility, with adjusted preload.** Intact muscle experiments performed as described in the main manuscript, with the following alterations. Single 1 Hz twitch activation was performed on both WT and Ttn^Δ219-225^ muscles at their respective L0. Next, the muscles were lengthened until they reached a baseline preload of 85 mg absolute force, for either genotype. This equalizes the passive stiffness between WT and Ttn^Δ219-225^ and allowed us to study the twitch response time in relation to the MyHC fiber type shift, without potential confounding effects of changes in titin-based stiffness.
